# Dual roles of HK3 in regulating the network between tumor cells and tumor-associated macrophages in neuroblastoma

**DOI:** 10.1007/s00262-024-03702-9

**Published:** 2024-05-07

**Authors:** Xin Wu, Tao Mi, Liming Jin, Chunnian Ren, Jinkui Wang, Zhaoxia Zhang, Jiayan Liu, Zhaoyin Wang, Peng Guo, Dawei He

**Affiliations:** 1 Department of Urology, Children’s Hospital of Chongqing Medical University, National Clinical Research Center for Child Health and Disorders, Ministry of Education Key Laboratory of Child Development and Disorders, Children’s Hospital of Chongqing Medical University, Chongqing, 400014 People’s Republic of China; 2 Chongqing Key Laboratory of Structural Birth Defect and Reconstruction, Chongqing, 400014 China; 3 Institute of Basic Medicine and Cancer (IBMC), Chinese Academy of Sciences, Hangzhou, 310022 Zhejiang China

**Keywords:** HK3, Neuroblastoma, M2-like macrophages, TAMs, TME, Pediatric solid tumor

## Abstract

Neuroblastoma (NB) is the most common and deadliest extracranial solid tumor in children. Targeting tumor-associated macrophages (TAMs) is a strategy for attenuating tumor-promoting states. The crosstalk between cancer cells and TAMs plays a pivotal role in mediating tumor progression in NB. The overexpression of Hexokinase-3 (HK3), a pivotal enzyme in glucose metabolism, has been associated with poor prognosis in NB patients. Furthermore, it correlates with the infiltration of M2-like macrophages within NB tumors, indicating its significant involvement in tumor progression. Therefore, HK3 not only directly regulates the malignant biological behaviors of tumor cells, such as proliferation, migration, and invasion, but also recruits and polarizes M2-like macrophages through the PI3K/AKT-CXCL14 axis in neuroblastoma. The secretion of lactate and histone lactylation alterations within tumor cells accompanies this interaction. Additionally, elevated expression of HK3 in M2-TAMs was found at the same time. Modulating HK3 within M2-TAMs alters the biological behavior of tumor cells, as demonstrated by our in vitro studies. This study highlights the pivotal role of HK3 in the progression of NB malignancy and its intricate regulatory network with M2-TAMs. It establishes HK3 as a promising dual-functional biomarker and therapeutic target in combating neuroblastoma.

**Supplementary Information:**

The online version contains supplementary material available at 10.1007/s00262-024-03702-9.

## Introduction

Neuroblastoma (NB) is the most frequent extracranial solid tumor in infants and young children, arising from primitive sympathetic nervous system precursor cells during embryonic development [1–3]. Globally, NB accounts for 6–10% of all pediatric malignancies, ranking highest in incidence among pediatric solid tumors [2]. The clinical presentation of NB varies significantly based on age, tumor location, and degree of tissue differentiation, predominantly characterized by infiltrative growth, indistinct margins, and difficulty in complete surgical removal [4]. Due to its resistance to radiochemotherapy and the significant side effects associated with high-intensity chemotherapy, the survival rate for NB patients remains at approximately 60%, notably lower than that observed for other common pediatric cancers, such as leukemia [5]. Neuroblastoma presents a substantial health threat to children and stands as a critical challenge in clinical treatment, emphasizing the pressing need to identify effective therapeutic targets for high-risk patients.

Tumor-associated macrophages (TAMs) are a fundamental stromal component within the tumor microenvironment (TME) [6]. They represent the most abundant myeloid cell population, exhibiting notable plasticity and functional diversity across different cancer types [6, 7]. A high density of TAMs is correlated with poor prognosis [8–10]. Research has identified that TAMs are predominantly classified into two phenotypes: the M1 anti-tumor phenotype and the M2 pro-tumor phenotype [11]. Additionally, TAMs are consistently induced to adopt an immunosuppressive M2-like phenotype in NB, thereby facilitating tumor progression and metastatic activity, consequently worsening the prognosis of high-risk NB [12]. In xenograft NB models, blocking macrophage stimulating factors such as CSF-1 has been demonstrated to prolong the survival of tumor-bearing mice [13]. Therefore, targeting TAMs and modulating their functionality to attenuate tumor-promoting actions might represent a promising strategy in future NB immunotherapy.

Metabolic reprogramming is a critical factor contributing to immune evasion by tumor cells within TME [14]. This process, which accelerates tumor cell proliferation and growth by modulating energy metabolism, originates from the Warburg effect [14]. Even in oxygen-rich conditions, tumor cells preferentially utilize aerobic glycolysis for rapid ATP production to support their proliferation, growth, and metastasis [15]. This reshaping of energy metabolism provides an advantage for tumor cell growth and proliferation, aids in evading apoptosis, and fosters TME more conducive to metastasis [14]. The interaction between malignant and immune cells within the TME synergistically promotes tumor development. Tumor cells can release cytokines like CSF1, IL-34, and VEGFA to induce metabolic reprogramming and phenotypic transition in TAMs like M2-like polarization [8]. For instance, in breast cancer, heightened glycolysis in tumor cells leads to a decrease in pH within the TME. This acidic environment activates signaling pathways in TAMs via G-protein-coupled receptors (GPCRs), ultimately driving their polarization toward the M2 phenotype [8]. Additionally, under hypoxic and lactate-stimulated conditions, TAMs can secrete cytokines such as IL-6, CCL5, and CCL18 to promote glycolysis in tumor cells [14, 16]. CCL5 and CCL18 upregulate various glycolysis-promoting factors, including hexokinase 2 (HK2), phosphoglycerate kinase 1 (PGK1), glucose-6-phosphate dehydrogenase (G6PD), and pyruvate kinase, further facilitating tumor progression [14, 16]. Exploring metabolic pathways in the interactions between tumor cells and TAMs holds significant clinical value for diagnosing and prognosing neuroblastoma patients.

Hexokinase-3 (HK3), a member of the hexokinase family, plays a crucial role in the early stages of glucose metabolism. Its gene is located on human chromosome 5q35.2 [17]. The mammalian hexokinase (HK) family, consisting of four structurally similar members (HK1-4), displays tissue-specific expression patterns [17]. Recent studies have shown significant upregulation of HK1 and HK2 in various malignant tumors, such as breast, colon, thyroid, and kidney cancers [18]. These enzymes regulate the glycolytic pathway, contributing to unfavorable prognostic outcomes [19, 20]. In colorectal cancer cell lines, HK1 and HK2 are dynamically regulated through feedback mechanisms [17]. Specifically, inactivation of HK2 results in increased HK1 expression. However, HK3 does not undergo similar feedback modulation by HK1 and HK2 [17]. Studies have shown that HK3 inactivation markedly disrupts glycolytic activation in colorectal cancer, initiating downstream signaling cascades such as apoptosis and endoplasmic reticulum stress. Therefore, HK3 plays a crucial role in the progression and evolution of cancer [20]. However, the precise role of HK3 in neuroblastoma progression and its interactions within the tumor microenvironment remains to be fully understood.

Our research aimed to elucidate the role of HK3 within the NB-TAMs regulatory network. These findings provide new biological insights into NB progression and may represent a promising therapeutic target for NB.

## Materials and methods

### Patient sample

The ten neuroblastoma samples for western blotting(2 cases for INSS I, 2 cases for INSS II, 2 cases for INSS III, and 4 cases for INSS IV, respectively) and 51 NB tissues for immunohistochemistry examining the expression of HK3 and CD163 were collected from Children's Hospital Affiliated with Chongqing Medical University. The histological diagnoses of all these samples were confirmed by two experienced pathologists. This study was approved by the Ethics Committee of the Children's Hospital Affiliated with Chongqing Medical University. Informed consent was obtained from each patient.

### Data download and processing

We obtained mRNA data and corresponding clinical information from the TARGET (Therapeutically Applicable Research to Generate Effective Treatments) database. To remove low-expressed mRNA, our methodology involved excluding samples with expression counts below 3 in 10% of the dataset.

### Prognostic analysis

For prognostic evaluation, univariate Cox regression analysis was carried out based on survival time and overall survival (OS) utilizing the survival package. A significance threshold of *P* < 0.05 was adopted to ascertain statistical significance.

### Gene set enrichment analysis (GSEA)

Gene set enrichment analysis (http://www.broadinstitute.org/gsea/index.jsp) was performed to explore whether the identified sets of genes showed statistical differences between the two groups stratified as described above.

### Quantification of immune infiltration

Immune cell profiling was performed using the Cibersort software package, leveraging gene sequencing data. The cytogenetic marker gene set employed was LM22, encompassing 547 genes associated with 22 distinct immune cell types. Analysis of variance (ANOVA) was applied to compare differences across groups, and a significance threshold of *P* < 0.05 was used to determine statistical significance.

### Western blot (WB)

Protein samples were lysed in RIPA buffer (Beyotime) supplemented with 1% protease inhibitor (Beyotime). The protein concentration was determined using the BCA assay kit (Beyotime). Equal protein amounts (10 μg) were subjected to 8% SDS-PAGE (sodium dodecyl sulfate–polyacrylamide gel electrophoresis) separation and transferred onto a polyvinylidene fluoride (PVDF) membrane. The membranes were then blocked using a rapid blocking solution and incubated with primary antibodies at 4 °C overnight. The following primary antibodies were employed: HK3 (1:1000, Proteintech), CD206 (1:1000, Proteintech), CD163 (1:1000, Proteintech), CXCL14(1:500, R&D), AKT (1:1000, Proteintech), P-AKT (1:1000, Proteintech), PCNA (1:1000, Proteintech), MMP2 (1:1000, Proteintech), MMP9 (1:1000, Proteintech), VEGF (1:1000, Proteintech), PI3K (1:1000, Proteintech), l-lactyl lysine (1:500, PTM) and GAPDH (1:1000, Proteintech). Subsequently, HRP-conjugated secondary antibodies (1:1000) were incubated at room temperature for 2 h. Visual immunoblotting was achieved using Immobilon Western chemiluminescent HRP substrate, and the density of GAPDH bands was quantified through analysis using Image Lab software(6.10, Bio-Rad).

### Immunohistochemistry (IHC) and immunofluorescence (IF)

Human tumor samples and mice tumor specimens were fixed using 4% paraformaldehyde (PFA). The process included paraffin embedding, sectioning, standard dewaxing, antigen retrieval, and a blocking step using 0.5% BSA. Primary antibodies were incubated overnight. Subsequently, secondary antibodies (1:200) were applied for a 30-min incubation at 25 °C. CD163, HK3, PCNA, VEGF, MMP9, and MMP2 expression levels were assessed through immunohistochemical (IHC) staining in 5 μm paraffin-embedded tissue sections. For immunofluorescence (IF), 4’,6-diamidino-2-phenylindole (DAPI, Solarbio) was used to counterstain the tissues. The Primary antibodies anti-CD163 (1:200, Proteintech), anti-HK3 (1:200, Proteintech), anti-PCNA (1:100, Proteintech), anti-MMP2 (1:200, Proteintech), anti-MMP9 (1:200, Proteintech)and anti-VEGF (1:200, Proteintech) antibodies were incubated overnight at 4 °C.The percentage or number of all positively stained cells was calculated as an average from six randomly selected microscope fields. The German Immunohistochemical Score (GIS) was utilized for the immunohistochemical assessment of paraffin-embedded sections [21]. The percentage of positive cells was graded as follows: 0 (negative), 1 (up to 10%), 2 (11–50%), 3 (51–80%), or 4 (greater than 80% positive cells). Staining intensity was graded as 0 (unstained), 1 (weak), 2 (moderate), or 3 (strong). The final immunoreactivity GIS was calculated as the product of the two grading results (percentage of positive cells * staining intensity). Low expression was defined as a GIS score of 6 or below, whereas high expression was determined as a score exceeding 6. The slides were scored independently by two experienced pathologists.

Co-cultured M0 Cells were harvested and washed in PBS. Then the sections were permeabilized with 0.5% Triton X-100 (Solarbio) for 20 min. After 5% BSA incubation for 1 h, sections were incubated in CD163 and CD68 at 4 °C overnight. Following incubation with fluorescein (FITC) or Cyanine3 (CY3) secondary antibody (Solarbio) and 4’,6-diamidino-2-phenylindole (DAPI, Solarbio), the samples were detected using a fluorescence microscope (Cannon). The images were merged digitally to monitor the co-localization condition.

### Cell lines and reagents

The SK-N-SH (Non-MYCN amplification) and SK-N-BE(2) cell line (MYCN amplification) (obtained from the Shanghai Institute of Biological Cell Research, Chinese Academy of Sciences) was cultured in DMEM medium supplemented with 10% fetal bovine serum (FBS; Gibco) [22, 23]. Additionally, the THP-1 cell line (also obtained from the Shanghai Institute of Biological Cell Research, Chinese Academy of Sciences) was cultured in 1640 medium supplemented with 10% fetal bovine serum (FBS; Gibco) and 100 U/ml penicillin–streptomycin. HUEVC Human vascular endothelial cells were isolated by collagenase digestion of the human umbilical cord veins obtained from Children's Hospital Affiliated with Chongqing Medical University with the informed consents signed by donors. Human vascular endothelial cells were cultured in Endothelial Cell Medium (Lonza). 0.99 μm BKM 120 for SK-N-SH, 1.2 μm BKM 120 or SK-N-BE(2) (one of a PI3K inhibitor) (MCE)was used for the PI3K-AKT experiment [24]. For THP-1 cells, 100 ng/ml of PMA (Phorbol 12-myristate 13-acetate) (Multi-Science) was added, and recombinant human CXCL14 (MCE) was used for the experiment in vitro [25].

### Lentivirus infection

A stable transfected SK-N-SH and SK-N-BE(2) cell line was engineered by utilizing lentiviral vectors. Human sh-HK3 plasmid and its corresponding negative control (vector) plasmid lentivirus mixtures were constructed by Genechem. These mixtures were subsequently co-cultured with NB cells, facilitating the establishment of a stable sh-HK3 SK-N-SH and SK-N-BE(2)cell line. The design of shRNA was based on the HK3 sequence (NM_002115.3), and the following sequence was employed: Sh-HK3-1: CGGAATGCGATGTCTCCTTAA, Sh-HK3-2: GCTTCGGATGTTGAGCTTGT, Sh-HK3-3: GCTGACCACCTTCGACCATAC. SK-N-SH and SK-N-BE(2) cells were transfected with both the Sh-con and Sh-HK3 constructs and the establishment of shRNA and sh-HK3 SK-N-SH and SK-N-BE(2) cell lines was achieved following a 3-week puromycin screening with a concentration of 2 μg/ml. The efficiency of HK3 knockdown was assessed at the protein level by Western blotting and PCR analysis.

### Animal experiments

A suspension of 1 × 10^6 tumor cells with sh-con/sh-HK3 was subcutaneously injected into the right axillary area of SPF-grade, 3-week-old male and female BALB/c mice (*n* = 12) in equal numbers. Tumor volume was measured every 2 days after tumor formation. Two weeks later, the mice were sacrificed under anesthesia. Tumor weight was measured. Tumor size was calculated using the formula: volume = (long diameter) × (short diameter)^2/2. A subset of tumor tissues was collected for subsequent analysis. The tumor tissues were embedded in paraffin, and immunostaining was performed to detect relevant markers and indexes.

Orthotopic NB models derived from SK-N-SH cells were established in the left adrenal gland using transfected SK-N-SH cells and M0 derived from THP-1, which were co-cultured with transfected SK-N-SH cells. To eliminate the faded role of intrinsic macrophages in mice, we injected clodronate liposomes/PBS liposomes (Liposoma BV) intraperitoneally into 3-week-old BALB/c mice every 2 days for 2 weeks to deplete the intrinsic macrophages in mice. Next, we transplanted 1 × 10^6 sh-con/sh-HK3 SK-N-SH cells, with or without 1 × 10^6 M0 cocultured with transfected SK-N-SH for 48 h. PBS liposomes were administered as a negative control. To maintain macrophage depletion in mice, the mice were injected with clodronate liposomes/PBS liposomes twice a week for 2 weeks. Mice were randomly assigned into 6 groups (*n* = 5 per group): 2 groups with PBS liposomes (sh-HK3 NB cells and sh-con NB cells) and 4 groups with clodronate liposomes (sh-HK3 NB cells, sh-con NB cells, sh-HK3, and sh-con NB cells co-cultured with M0 for 48 h, respectively). Two weeks post-transplantation, mice were euthanized, and tissues from the lungs, spleen, liver, kidney, and tumor were harvested for comprehensive pathological examination. To prevent fragmentation of the tumor during its separation, we first photographed the kidney and adrenal tumor together as a whole. Subsequently, the tumor was carefully dissected for the precise measurement of its weight and volume. Tumor size was calculated using the formula: volume = (long diameter) × (short diameter)^2/2. All animal experiments were approved by the Institutional Animal Care and Use Committee of the Children's Hospital Affiliated with Chongqing Medical University.

### M0 macrophage induction

THP-1 cells exhibiting robust growth were harvested. These cells were suspended using 1640 medium without fetal bovine serum (FBS) and supplemented with a concentration of 100 ng/ml of PMA (Phorbol 12-myristate 13-acetate). Subsequently, 1640 medium without FBS was added to generate a cell suspension with a concentration of 1 × 10^6 cells/ml. This suspension was evenly distributed within 10 cm^2 culture dishes for cultivation. Following an incubation period of 48 h, the cellular state was examined. Successful induction of M0 tumor-associated macrophages from THP-1 cells was confirmed if a significant proportion of cells transitioned from a suspended state to adherent growth. Additionally, observation of a few cells exhibiting pseudopodia formation further affirmed the successful induction process.

### Cell co-culture

Sh-HK3/sh-con SK-N-SH and SK-N-BE(2) cells at a concentration of 1 × 10^5 cells in 1 ml were introduced into the upper chamber of a Falcon compartment equipped with a Labselect 3.0 μm membrane. These cells were co-cultured with M0 macrophages, also at a concentration of 1 × 10^5 cells in 1 ml, positioned in the lower chamber. This co-culture scenario was sustained for 48 h. For the control group, M0 macrophages at a concentration of 1 × 10^5 cells in 1 ml were individually incubated with 10% FBS RPMI 1640 medium. In specific experiments, Recombinant human recombinant CXCL14 protein at a concentration of 20 and 100 ng/ml was introduced to the co-culture system and maintained for an additional 48 h to facilitate subsequent experimental analysis.

### Flow cytometry

THP-1 macrophages were co-cultured with sh-con/sh-HK3 SK-N-SH cells for 48 h, both in the presence and absence of Recombinant human CXCL14 protein (MCE). Subsequently, these macrophages were processed into single-cell suspension and treated with 250 μL of Fixation/Permeabilization solution for 20 min at 4 °C. The cells were then washed with 1 × BD Perm/Wash TM buffer, then incubated with APC Mouse anti-Human CD163 antibodies (BD Biosciences) and BV421 Mouse anti-Human CD68 antibodies (BD Biosciences). This incubation was conducted for 30 min at 4 °C. Flow cytometry was analyzed using a FACS Calibur flow cytometer from BD Biosciences. The acquired data was subjected to flow cytometric analysis using FlowJo software (version 10.4, USA).

### Wound healing and Transwell assay

SK-N-SH and SK-N-BE(2) cells were seeded in a 6-well plate at a density of 2 × 10^5 cells per well. The cells were cultured until they completely covered the surface of the well. A vertical scratch was created across the confluent monolayer using a sterile instrument. Following the scratching, the cellular debris was washed away with PBS, and the cells were subjected to serum-free DMEM culture. The progress of scratch closure was observed under an inverted microscope, and images were captured at 0, 24, and 48 h.

For the migration assay involving SK-N-SH/SK-N-BE(2), cells co-cultured with M0 macrophages, and single-cell suspensions were prepared. These cells were suspended in a serum-free DMEM medium. A 500 μL cell suspension containing 20,000 cells was added to the upper chamber of transwell inserts featuring an 8 μm microporous membrane (Corning). Before cell addition, the transwell chamber's basement membrane was pre-coated with a 1:8 matrix gel (Corning). Subsequently, 600 μL of DMEM medium containing 10% FBS was added to the lower chamber of the transwell inserts. Following 24 and 48 h of standard culture, the cells underwent three washes using PBS. They were then fixed with 4% paraformaldehyde for 30 min. Crystal violet (Solarbio) staining was performed for an additional 30 min, followed by the removal of excess dye using a cotton swab. Five random visual fields were selected for imaging and cell counting using a microscope after drying the basement membrane. This experimental procedure was repeated three times to ensure reliability and reproducibility.

### Macrophage migration assay

THP-1 cells were polarized in vitro. Following successful polarization, sh-HK3 and sh-con SK-N-SH/SK-N-BE(2) cells (1 × 10^5 cells/mL) were seeded into the upper chamber of a Falcon chamber (Labselect, 3.0 μm), while M0 macrophages (1 × 10^5 cells/mL) were co-cultured in the lower compartment for 48 h.

Subsequently, the sublayer of macrophages was collected and placed into the upper layer of transwell inserts featuring an 8 μm microporous membrane. Before cell addition, the transwell basement membrane was pre-coated with a 1:8 matrix gel. Then, 600 μL of 1640 medium containing 10% FBS was added to the lower chamber of the transwell inserts. After 24 and 48 h of standard culture, the cells underwent three washes using PBS. They were then fixed with 4% paraformaldehyde for 30 min. After fixation, the cells were stained with crystal violet (Solarbio) for 30 min. Subsequently, a cotton swab was used to remove excess dye. After the basement membrane was dry, five random visual fields were selected for imaging and cell counting using a microscope (Canon). This entire experiment was repeated three times to ensure statistical reliability and reproducibility.

### Analysis of lactate levels

The levels of lactate in NB cell culture supernatant were measured by Lactate Assay Kit (Applygen), by the manufacturer’s Research. The assay was repeated at least three times.

### Enzyme-linked immunosorbent assay (ELISA)

The cytokine of CXCL14 in cell supernatants was estimated by ELISA, using a commercial kit (Multi-Sciences), according to the manufacturer’s instructions. Positive controls were supplied in the kit.

#### RT-PCR

Total RNA was extracted from M0 cells, and M0 cells co-cultured with transfected NB for 48 h. The extracted RNA was then reverse transcribed into cDNA using the mRNA Reverse Transcription First Strand Kit (Takara), following the manufacturer's instructions. The mRNA expression levels were assessed using the mRNA RT-PCR Assay Kit (Abclonal). Primers used for amplification are provided in Table 1.

**Table 1:** The primer sequence used in this article

Name	Primer sequence (5’-3’)	TM (℃)
CD163	Forward: ATCAACCCTGCATCTTTAGACA	60
	Reverse: CTTGTTGTCACATGTGATCCAG	
CD206	Forward: GACGTGGCTGTGGATAAATAAC	60
	Reverse: CAGAAGACGCATGTAAAGCTAC	
CD68	Forward: CCCAGATTCAGATTCGAGTCAT	60
	Reverse: GTTTTGTTGGGGTTCAGTACAG	
HK3	Forward: GGGTGACTCTAACTGGCATTGA	60
	Reverse: TGACAAGGGAAAGAGAAGCTGAA	
IL-10	Forward: AGCTCCAAGAGAAAGGCATCTAC	60
	Reverse: GTCTATAGAGTCGCCACCCTGAT	
CCL18	Forward: GGTGTCATCCTCCTAACCAAGAG	60
	Reverse: GGCATAGCAGATGGGACTCTTAG	
IL-4	Forward: AGTTCTACAGCCACCATGAGAAG	60
	Reverse: GTACTCTGGTTGGCTTCCTTCAC	
CXCL14	Forward: ACTGCGAGGAGAAGATGGTTATC	60
	Reverse: CAGGCGTTGTACCACTTGATGAA	

### Statistical analysis

Statistical analyses were conducted using GraphPad Prism software (Version 9.4.1, USA). The relationship between CD163 expression and HK3 expression in NB patients was evaluated using Pearson's correlation analysis. Comparison of discrete variable groups was performed using either Student's *t* test or nonparametric ANOVA, depending on the nature of the data. Each experiment was conducted independently a minimum of three times. Results are presented as means ± SEM. In in vivo experiments, data are also expressed as mean ± SEM. Statistical significance was determined using *p*-values < 0.05 (*p < 0.05; **p < 0.01; ***p < 0.001; ****p < 0.0001), which were considered statistically significant in all figures.

## Results

### HK3 overexpression predicts a poor prognosis in neuroblastoma patients

**Fig. 1:**
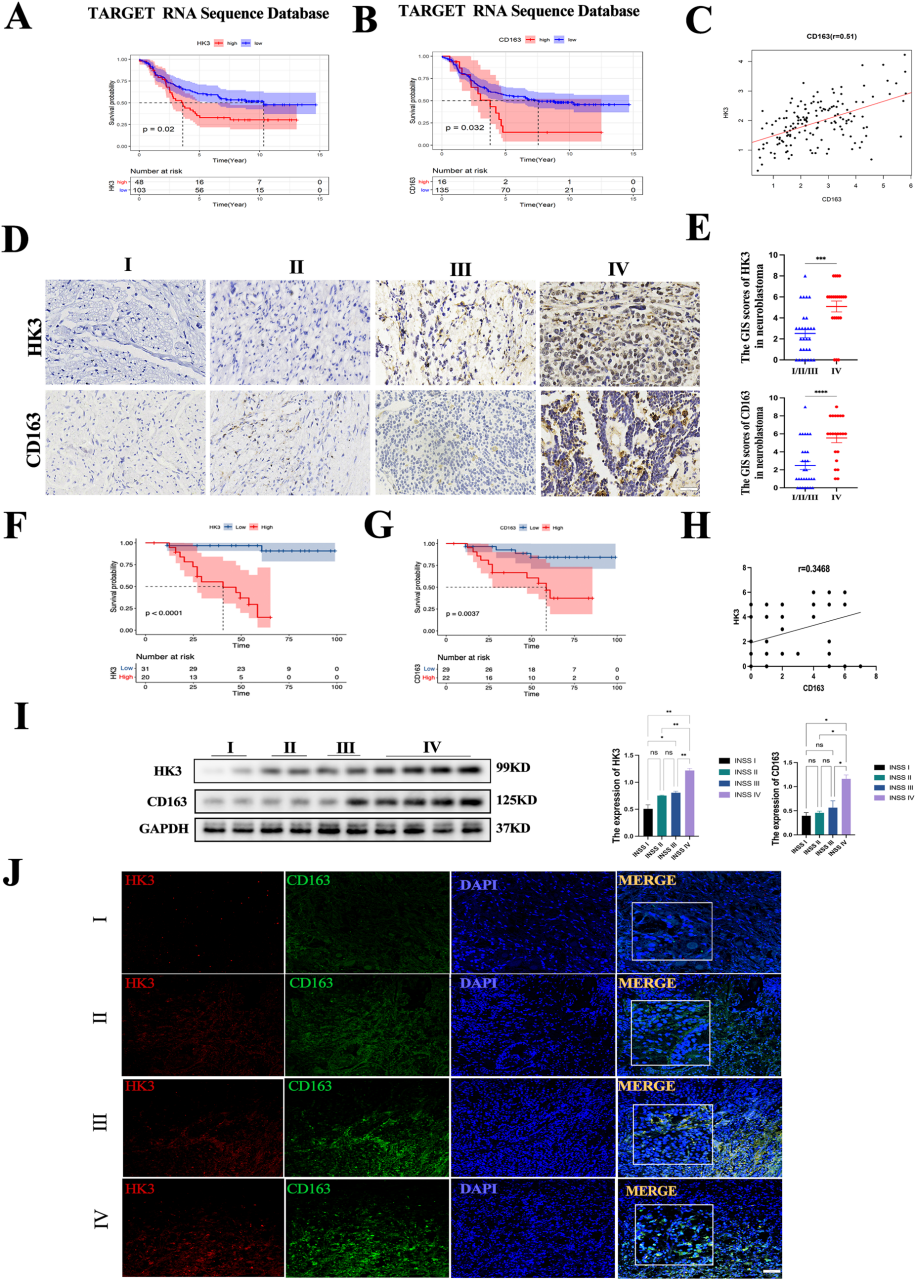
Overexpression of HK3 indicated a poor prognosis in neuroblastoma patients and high M2 macrophage infiltration in NB. **a** Kaplan–Meier curves for Overall Survival (OS) demonstrated that patients with high HK3 expression (*n* = 48) exhibited a significantly poorer prognosis in the TARGET database (*P* = 0.02). **b** Kaplan–Meier curves for Overall survival (OS) showed that CD163-high patients (*n* = 16) exhibited a significantly poorer prognosis in the TARGET database (*P* = 0.032). **c** The correlation between HK3 and CD163 in the TARGET RNA sequence database (*r* = 0.51). **d** IHC staining of HK3 and CD163 in clinical tumor samples with different INSS stages, scale bar = 100 μm. **e** The GIS scores graph of IHC staining for HK3 and CD163 in clinical Neuroblastoma tissues of INSS stage IV compared to INSS stage I/II/III (*n* = 51). **f** The Kaplan–Meier curves for Overall Survival (OS) revealed that HK3-high patients (*n* = 20) demonstrated a poorer prognosis compared to HK3-low patients (*n* = 31) based on IHC staining in clinical samples (*n* = 51) (*P* < 0.0001). **g** Kaplan–Meier curves for Overall Survival (OS) indicated that CD163-high patients (*n* = 22) experienced a poorer prognosis compared to CD163-low patients (*n* = 29) based on IHC staining in clinical samples (*n* = 51) (*P* < 0.0037). **h** The correlation between HK3 and CD163 of IHC staining in clinical samples (*r* = 0.3468). **i** Western blotting of HK3 and CD163 in clinical tissues with different INSS stages (*n* = 10). **j** Immunofluorescent staining of the co-expression level of HK3 (red) and CD163 (green) in different INSS stages of NB tissues. DAPI (blue) stained for nuclei, scale bar = 100 μm (ns, no significance; *p < 0.05; **p < 0.01; ***p < 0.001, ****p < 0.0001)

First, the clinical relevance of HK3 overexpression and clinical outcomes was investigated in NB patients. By querying 151 NB samples in the TARGET database, Kaplan–Meier survival analysis showed that HK3-high patients had a significantly poorer prognosis (*P* < 0.05, Fig. 1a). Furthermore, three datasets containing NRC-283 (2,888,485) HK3, Kocak-476 (ukv A24) HK3, and Vesteeg-88 (205,936-s-at) HK3 were also examined in R2 (https://hgserver1.amc.nl/cgi-bin/r2/main.cgi), yielding similar results (Supplementary Fig. 1a–c). These trends were subsequently verified at the protein level in 51 pathological sections and 10 fresh tissue samples collected from the Children’s Hospital of Chongqing Medical University using immunohistochemistry and western blotting. It was observed that HK3 levels were consistently upregulated in INSS stage IV compared to other stages (*n* = 10, Fig. 1d, i. Based on HK3 expression by IHC, patients were divided into HK3-high (*n* = 20, 39%) and HK3-low (*n* = 31, 61%) groups. In consistency with mRNA expression from the TARGET database, Kaplan–Meier survival analysis also showed that HK3-high patients had poorer outcomes than HK3-low patients (*P* < 0.05, Fig. 1f). The clinicopathological analysis indicated INSS, Shimada, Risk, the expression of CD163, and Marrow metastasis were also associated with HK3 expression (Pearson’s chi-square test, *P* < 0.05). Due to limited sample sizes in INSS stages I, II, and III, we merged these stages for statistical analysis. In contrast, age, MYCN, bone metastasis type, and gender exhibited no significant correlation with HK3 expression (Table 2). Multivariate Cox regression analysis suggested that HK3 expression and INSS were independent prognostic factors in NB patients (Table 3). Considering the unclear role of HK3 in NB progression, HK3 was selected for further exploration.

**Table 2:** The clinicopathological characteristics based on HK3 expression

	*N* = 51	HK3 low (*N*= 31)	HK3 high (*N* = 20)	*P*
Tumor size				0.532
< 10 cm	27 (52.9%)	18 (58.1%)	9 (45.0%)	
≥ 10 cm	24 (47.1%)	13 (41.9%)	11 (55.0%)	
Age				0.312
< 18 m	21 (41.2%)	15 (48.4%)	6 (30.0%)	
≥ 18 m	30 (58.8%)	16 (51.6%)	14 (70.0%)	
Gender				0.080
Female	19 (37.3%)	15 (48.4%)	4 (20.0%)	
Male	32 (62.7%)	16 (51.6%)	16 (80.0%)	
INSS				
1/2/3	23 (55.1%)	22 (95.65%)	1(4.35%)	< 0.001
4	28 (54.9%)	9 (32.14%)	19 (67.86%)	
Risk				< 0.001
Non-high risk	21 (41.2%)	19 (61.3%)	2 (10.0%)	
High risk	30 (58.8%)	12 (38.7%)	18 (90.0%)	
Shimada				0.033
Fh	18 (35.3%)	15 (48.4%)	3 (15.0%)	
uFh	33 (64.7%)	16 (51.6%)	17 (85.0%)	
MYCN				0.356
Non-amplify	38 (74.5%)	25 (80.6%)	13 (65.0%)	
Amplify	13 (25.5%)	6 (19.4%)	7 (35.0%)	
Marrow metastasis				0.039
No	33 (64.7%)	24 (77.4%)	9 (45.0%)	
Yes	18 (35.3%)	7 (22.6%)	11 (55.0%)	
Bone.metastasis				0.289
No	42 (82.4%)	27 (87.1%)	15 (75.0%)	
Yes	9 (17.6%)	4 (12.9%)	5 (25.0%)	
CD163				< 0.001
Low	29 (56.9%)	24 (77.4%)	5 (25.0%)	
High	22 (43.1%)	7 (22.6%)	15 (75.0%)

**Table 3:** Univariate multivariate Cox regression analysis

Variable		univariate			multivariate		
		Hazard ratio	95%CI	*P*	Hazard ratio	95%CI	*P*
Tumor size							
	< 10 cm	1			1		
	≥ 10 cm	1.11	0.4–3.07	0.839		0.727–1.221	0.65
Age							
	< 18 m	1			1		
	≥ 18 m	1.12	0.38–3.28	0.838	0.942	0727–1.221	0.063
Gender							
	Male	1			1		
	Female	2.61	0.73–9.26	0.139	0.968	0.212–4.424	0.966
INSS							
	1/2/3	1			1		
	4	7.89	2.21–22.79	0.01	5.69	1.352–23.955	0.018
Risk							
	High-risk	1			1		
	Non-high risk	5.13	1.16–22.79	0.032	6.802	0.673–68.745	0.104
Shimada							
	Fh	1			1		
	Ufh	4.03	0.91–17.96	0.067	0.959	0.139–6.627	0.967
MYCN							
	Non-amplify	1			1		
	Amplify	1. 71	0.58–5.01	0.331	0.284	0.042–1.901	0.194
Marrow metastasis							
	No	1			1		
	Yes	3.38	1.19–9.56	0.022	1.187	0.384–9.116	0.438
Bone metastasis							
	No	1			1		
	Yes	1.56	0.44–5.55	0.495	0.459	0.082–2.573	0.495
HK3							
	Low	1			1		
	High	21.89	4.64–103.16	0.001	21.042	3.679–120.343	0.018
CD163							
	Low	1			1		
	High	4.7	1.49–14.81	0.008	1.521	0.363–6.377	0.567

### M2 macrophage is highly infiltration in neuroblastoma tumors and is strongly associated with HK3

Considering immune cells exert critical functions in promoting NB tumor development and progression, the immune cells in the NB were subsequently investigated. A total of 22 types of immune cells were quantified for their NB tumor infiltration from the TARGET database using CIBERSORT. A significant abundance of M2-like macrophages within the tumor was observed (Supplementary Fig. 1d). Kaplan–Meier analysis revealed that patients with high M2-like macrophage infiltration also had a poor prognosis in the TARGET database (Fig. 1b). This association was also evident for the M2-like macrophage marker CD163 (Supplementary Fig. 1e). The same trends in the tumor sample were verified by IHC and WB (*n* = 10, Fig. 1d). The Western blot results showed elevated CD163 expression in NB tumors at INSS stage IV compared to INSS I/II/III in 10 tumor tissues (*n* = 10, Fig. 1i). Kaplan–Meier analysis revealed that patients with high CD163 expression had a poor prognosis in the tumor detected by IHC (*P* < 0.001, Fig. 1g).

To further investigate the relationship between HK3 and M2-like macrophages, RNA sequencing from the TARGET database and experimental results were conducted. A significantly positive correlation between HK3 expression and M2-like macrophage expression, as well as the M2-like macrophage marker CD163 was found (Supplementary Fig. 1f, *r* = 0.31; Fig. 1c, h). Moreover, immunofluorescence staining of NB tissues suggests that cells positive for HK3 co-express with cells positive for CD163 in the same region, and the co-expression level of HK3 and CD163 increased in INSS IV (Fig. 1j), indicating that the number of infiltrated CD163 macrophages have a positive correlation with HK3 level in NB. Together, these results suggest that HK3 is closely associated with NB progression and M2-like macrophage infiltration, leading to unsatisfactory outcomes.

### Neuroblastoma-intrinsic HK3 mediates proliferation, migration, and invasion

To investigate the role of HK3 in NB cells, the expression of HK3 in NB cell lines, and human umbilical vein endothelial cells (Huvec, a type of cell within the tumor microenvironment) as control was tested by RT-PCR. We found HK3 was enriched in all NB cell lines, especially in SK-N-SH and SK-N-BE(2)than in umbilical vein endothelial cells (Supplementary Fig. 2a). These two cell lines respectively represent the MYCN amplified and non-amplified states of neuroblastoma [22, 23]. Next, the effect of HK3 knockdown on the malignant biological behavior phenotype of neuroblastoma was investigated in vitro. Stable transfections were established with sh-HK3-1, sh-HK3-2, and sh-HK3-3. Sh-HK3-1 showed minimal green fluorescence expression after transfection and was excluded. Sh-HK3-3 had the highest efficiency of knockdown, which was approximately 30% of the sh-con group (Supplementary Fig. 2b, c). This sequence was chosen for subsequent experiments.

**Fig. 2:**
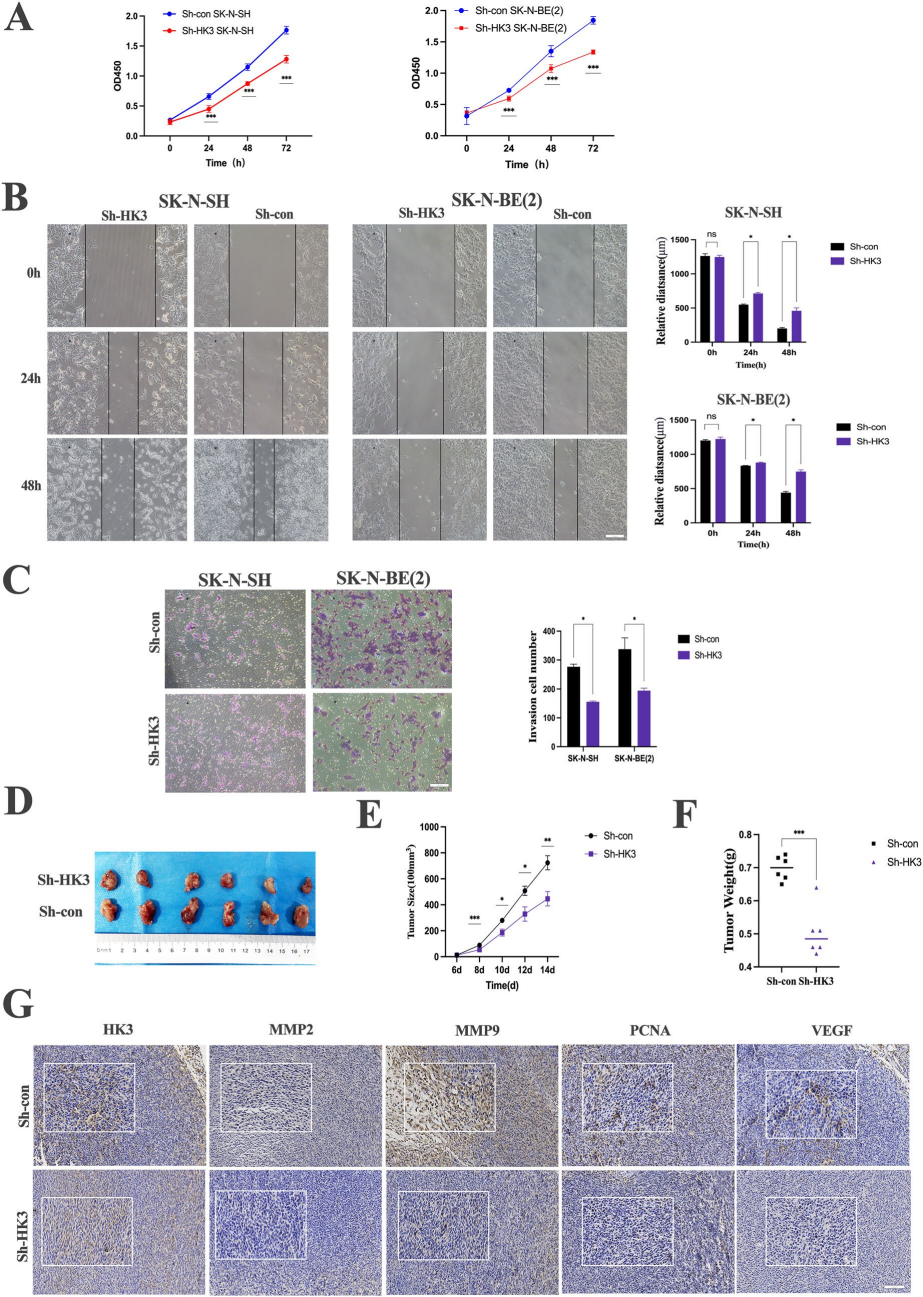
Neuroblastoma-intrinsic HK3 mediates proliferation, migration, and invasion. **a** Cell viability assay. Knock-down HK3 in SK-N-SH and SK-N-BE(2) decreased the proliferation of stably transfected cells. **b** Wound-healing assay. The data showed that HK3 silence reduced the tumor cell migration ability of SK-N-SH and SK-N-BE(2), scale bar = 100 μm. **c** Transwell assay. Knock-down HK3 in SK-N-SH and SK-N-BE(2) decreased the invasion ability of stably transfected cells, scale bar = 100 μm. **d** Subcutaneous xenograft tumor model. Macroscopic images of tumor tissues from sh-HK3 and sh-con SK-N-SH cells. **e**–**f** Quantitative analysis of tumor size (**e**) and tumor weight (**g**) Immunohistochemistry assay. IHC staining of tumors in tumor-bearing mice after SK-N-SH with HK3 knockdown or negative control implanted subcutaneously, scale bar = 100 μm (ns, no significance; *p < 0.05; **p < 0.01; ***p < 0.001, ****p < 0.0001)

Subsequently, the impacts of HK3 on NB cell proliferation, migration, and invasion were investigated. Our data indicated that downregulation of HK3 expression reduced cell viability in stable transfected SK-N-SH and SK-N-BE(2) cells (*p* < 0.05) as shown in Fig. 2a. Wound healing experiments (Fig. 2b) and transwell assay (Fig. 2c) revealed that HK3 knockdown reduced the migration and invasion abilities of SK-N-SH and SK-N-BE(2) cells than the control group.

Next, a subcutaneous xenograft tumor model was used to identify the effect of HK3 knockdown on the malignant biological phenotype of neuroblastoma in vivo. SK-N-SH cell lines transfected with sh-HK3/sh-con were transplanted into 4-week-old BALB/C mice to establish a subcutaneous xenograft tumor model. 2 weeks later, mice were killed and dissected. The sh-HK3 group exhibited significant reductions in tumor volume and weight compared to the sh-con group, indicating statistical significance. (Fig. 2d-f). Throughout the experiment, no distance metastasis of the tumor was observed during necropsy. IHC analysis of tumors derived from mice was also performed to assess relevant malignancy. It was observed that the expression of PCNA, MMP2, and MMP9 was significantly decreased in the HK3 knockdown group of NB tumors while the expression of VEGF showed a slight decrease (Fig. 2g). These data highlight the important role of neuroblastoma intrinsic HK3 in promoting the tumorgenicity of neuroblastoma cells.

### Inhibition of M2 macrophage polarization co-cultured with neuroblastoma cells after knockdown of HK3 expression

**Fig. 3:**
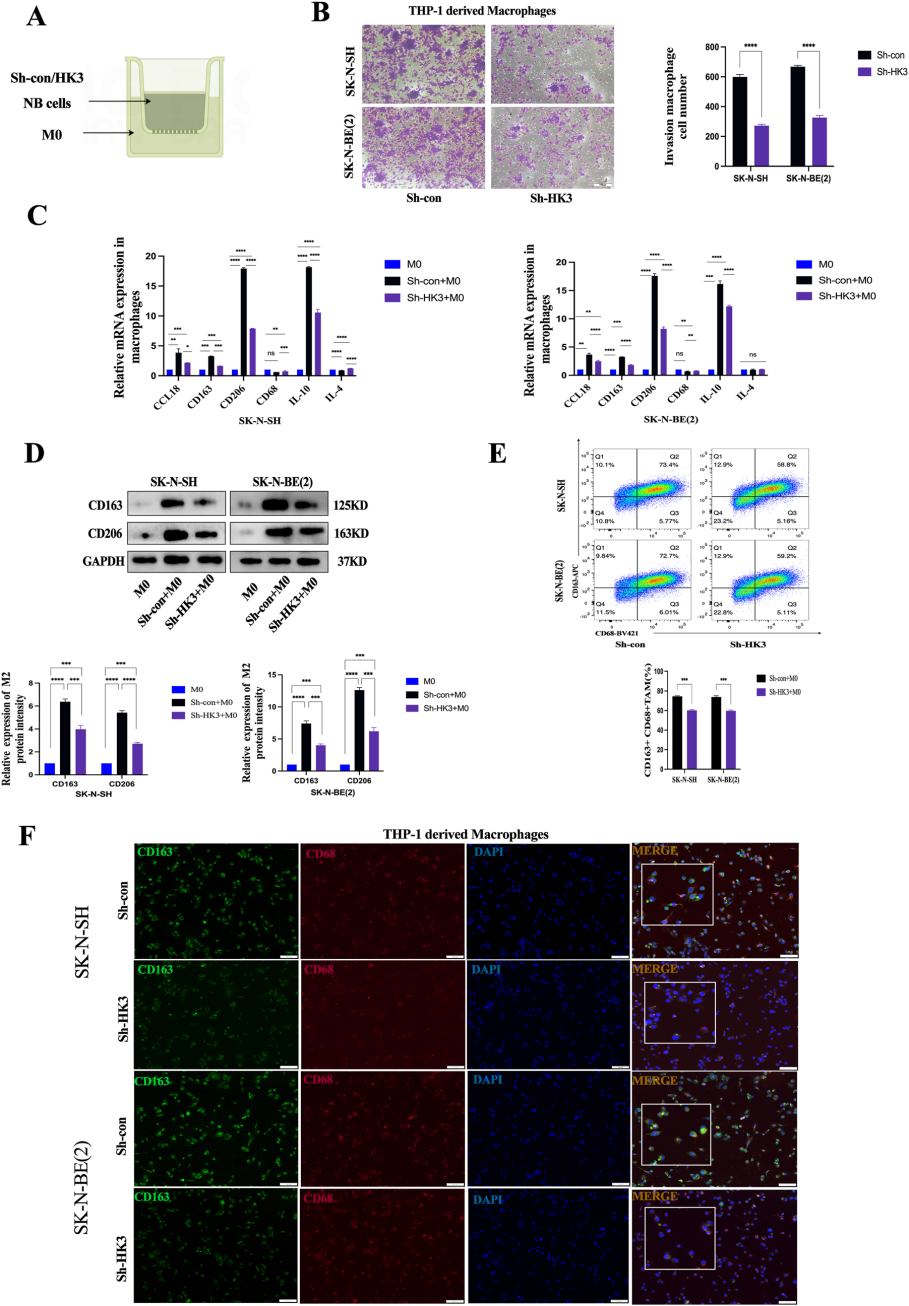
Inhibition of M2 macrophage polarization co-cultured with neuroblastoma cells after knockdown of HK3 expression. **a** Schema for an in vitro model of transfected NB cells co-cultured with M0. **b** Macrophage migration assay. The migration and invasion ability of M2 macrophage cells after co-culturing with sh-con/sh-HK3 SK-N-SH and SK-N-BE(2) for 48 h, scale bar = 100 μm. **c** RT-PCR. The expression of several cytokines in macrophages co-cultured with sh-con/sh-HK3 SK-N-SH and SK-N-BE(2) for 48 h (Student’s *t*-test, *n* = 3). **d** Western blot. The protein expression of M2 marker CD163 and CD206 in M0 and macrophage after co-culturing with sh-con/sh-HK3 SK-N-SH and SK-N-BE(2) for 48 h. **e** Flow cytometry was used to explore the surface expression of CD68 and CD163 in macrophage cocultured with sh-con/sh-HK3 SK-N-SH and SK-N-BE(2) cells. error bars, SEM. **f** Dual Immunofluorescence of CD163 and CD68 in macrophages co-cultured with sh-con/sh-HK3 SK-N-SH and SK-N-BE(2) cells for 48 h, scale bar = 100 μm (ns, no significance; *p < 0.05; **p < 0.01; ***p < 0.001, ****p < 0.0001)

Considering the correlations observed in our prior investigations between HK3 and the M2 macrophage marker CD163 in clinical samples and TARGET databases, the interplay between HK3 and M2 macrophages was subsequently explored. This endeavor involved the establishment of an in vitro co-culture system involving NB cells and macrophages (Fig. 3a). It was observed that THP-1 cells underwent induction from suspension to adherent growth upon incubation with PMA, accompanied by the cessation of proliferation and a morphological transition from round to spindle or irregular shapes (Supplementary Fig. 3a). Our initial inquiry focused on whether HK3 could influence macrophage recruitment. Through the transwell assay, following 48 h of co-culturing sh-con/sh-HK3 NB cells with M0 macrophages, a decrease in the recruitment capacity of M2-like macrophages was observed within the HK3-knocked down group (Fig. 3b). The cytokines of the M2 phenotype (CD206, CD163, CCL18, IL-4, and IL-10) were investigated using qPCR assays. Compared to M0 macrophages, TAMs exhibited predominantly increased expression of M2 markers after co-culture with stably transfected NB cells (Fig. 3c). M0 macrophages co-cultured with sh-HK3 NB cells displayed a notable decrease in CD206 and CD163 expression, along with related cytokines such as CCL18 and IL-10. Meanwhile, the expression of the pan-macrophage marker CD68 showed minimal changes (Fig. 3c). Western blot analyses revealed a reduction in CD163 and CD206 expression in macrophages co-cultured with sh-HK3 neuroblastoma cells (Fig. 3d). Flow cytometry analysis demonstrated a significant decrease in the double-positive expression of CD68 and CD163 in macrophages after co-culture with the knockdown of HK3 NB group (Fig. 3e). The immunofluorescence staining results indicated a significant reduction in the expression levels of CD163 in macrophages co-cultured with HK3-knockdown neuroblastoma cell lines, compared to the control group. At the same time, there were no apparent changes observed in the expression of CD68 (Fig. 3f). Collectively, these findings underscore the pivotal role of HK3 in orchestrating M2-like macrophage recruitment and polarization within the context of neuroblastoma.

### Tumoral HK3 orchestrates tumorigenesis and invasion by modulating M2 macrophages in vivo

**Fig. 4:**
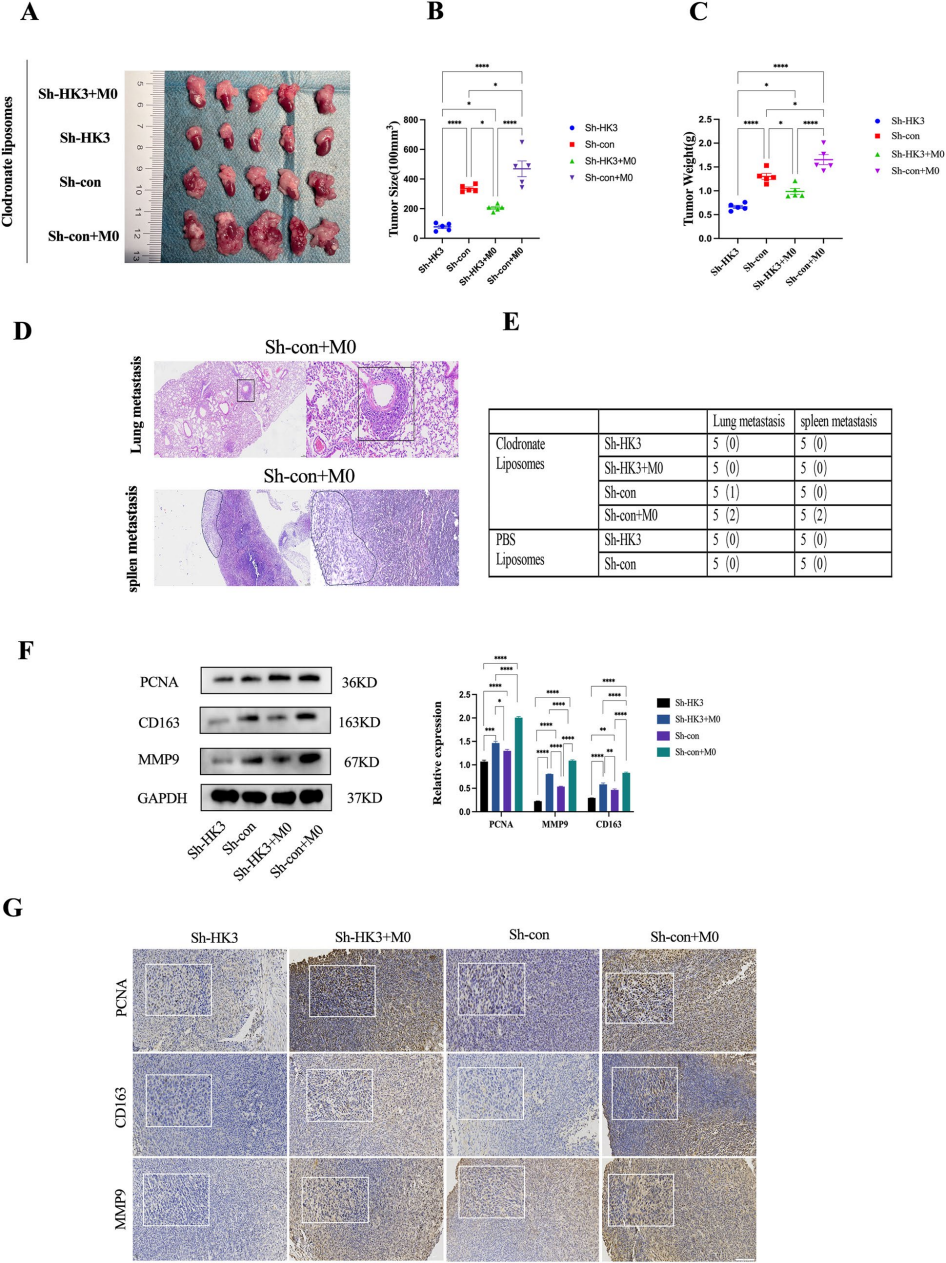
Tumoral HK3 orchestrates tumorigenesis and invasion by modulating M2 macrophages in vivo. **a** Orthotopic xenograft tumor model. Macroscopic images of kidney and tumor tissues of 4 Clodronate liposomes transplant group (sh-HK3, sh-con, sh-con + M0, and sh-HK3 + M0 SK-N-SH cells). **b–c** Quantitative analysis of tumor size (**b**) and tumor weight (**c**). **d** The H&E staining of sh-con + M0 transplant group. The area outlined in black exhibits pronounced tumor cell infiltration, lung metastasis (left scale bar = 50 μm; right scale bar = 10 μm), and spleen metastasis (left scale bar = 100 μm; right scale bar = 25 μm). **e** Statistical plot of tumor metastatic sites in mice. **f** Western blot. The expression of PCNA, CD163, MMP9, and GAPDH protein in 4 clodronate liposomes transplant group (sh-HK3, sh-con, sh-con + M0, and sh-HK3 + M0 SK-N-SH cells). **g** Immunohistochemistry assay. The expression of PCNA, CD163, and MMP9 protein in 4 clodronate liposomes transplant group(sh-HK3, sh-con, sh-con + M0, and sh-HK3 + M0 SK-N-SH cells)scale bar = 100 μm ( ns, no significance; *p < 0.05; **p < 0.01; ***p < 0.001, ****p < 0.0001)

To figure out whether the crosstalk between NB-derived HK3 and M2-like macrophages influences tumorigenesis and malignancy, tumor growth, and metastasis in vivo by using cell line-derived orthotopic NB models were evaluated. The orthotopic model was established by left adrenal gland injection with stable transfected SK-N-SH cells. Considering the role of intrinsic macrophages in mice, clodronate liposomes/ PBS liposomes were intraperitoneally injected into BALB/c mice to deplete the intrinsic macrophages in mice. Immunohistochemical staining of the macrophage marker F4/80 confirmed the clearance of macrophages in major organs of the mice (Supplementary Fig. 4a). Subsequently, human sh-con/sh-HK3 SK-N-SH cells, co-cultured with or without THP-1-derived macrophages for 48 h at a 1:1 ratio, were then co-transplanted into mice. Next, the malignant effects of tumoral HK3 with tumor-associated macrophages in adrenal orthotopic neuroblastoma xenograft models were sought to be assessed. Results indicated that mice transplanted with HK3-knockdown SK-N-SH cells exhibited smaller tumor sizes and weight when treated with liposomes compared with the control group (Fig. 4a–c). After co-culturing with macrophages, both sh-HK3 and sh-con tumor cells showed increased tumor volume and weight compared to the group with only tumor cell transplantation (Fig. 4a–c). Simultaneously, tumor cells with HK3 knockdown, co-cultured with macrophages, showed significantly lower increases in tumor size and weight compared to the control group (Fig. 4a–c). Surprisingly, under conditions of mouse macrophage depletion, the control group of tumor cells co-cultured with macrophages exhibited 1 case of lung metastasis and 2 cases of spleen tumor infiltration, attributed to large tumor size (Fig. 4d, e). In the PBS liposomes control group, smaller tumor size and weight were observed compared to the clodronate liposomes group, with no significant distant metastatic foci or abdominal tumor spread noted. Under these conditions, HK3 also appears to exert an inhibitory effect on tumor growth (Supplementary Fig. 4b-d). Subsequently, IHC and western blot were utilized to assess the malignant phenotype and M2 macrophage markers under macrophage depletion conditions. The results revealed higher expression intensity of malignant markers PCNA, MMP9, and M2-TAMs marker CD163 in the co-transplantation of sh-con and macrophages compared to the other groups (Fig. 4f, g). The malignant phenotype of the group solely implanted with sh-con tumor cells was slightly lower than that of the co-culture group but higher than that of the group with sh-HK3 and macrophages co-cultured. The sole implantation of the sh-HK3 group exhibited the lowest malignancy (Fig. 4f, g). The macrophage marker CD163 exhibited a similar trend (Fig. 4f, g). Taken together, these findings support the notion that inhibiting HK3 in neuroblastoma, while simultaneously suppressing its interaction with macrophages, can effectively mitigate tumor tumorigenesis and progression. Moreover, the presence of macrophages co-cultured with tumor cells (M2-TAMs) can promote tumor progression and augment invasion and metastatic potential.

### HK3 in neuroblastoma affects lactate secretion in the microenvironment and regulates histone lysine lactylation

In our previous study, it was demonstrated that the polarization of M2 macrophages in the tumor microenvironment could be regulated by tumoral HK3 both in vitro and in vivo. The potential mechanisms of this phenotype were sought to be elucidated. Since HK3 is a subtype of hexokinase involved in the first step of glucose metabolism, lactate is a direct by-product of glycolysis, which has been widely demonstrated to be directly involved in tumor progression by M2 macrophage polarization in tumor microenvironment [18, 26]. Histone lysine lactylation (Kla) was identified as a novel post-modification (PTM) [27]. Numerous studies have demonstrated that lactate promotes the polarization of macrophages toward an M2-like phenotype through the lactylation of histone lysines [27, 28]. Therefore, the impact of HK3 knockdown on lactate levels and histone lactylation within the NB microenvironment was aimed to be explored. To accomplish this, the regulation of protein lactylation levels by HK3 was examined through Western blot analysis using an anti-l-Lactyl lysine antibody. Additionally, lactate levels in the cell supernatant of neuroblastoma cancer cells were assessed using a Lactate Assay Kit.

**Fig. 5:**
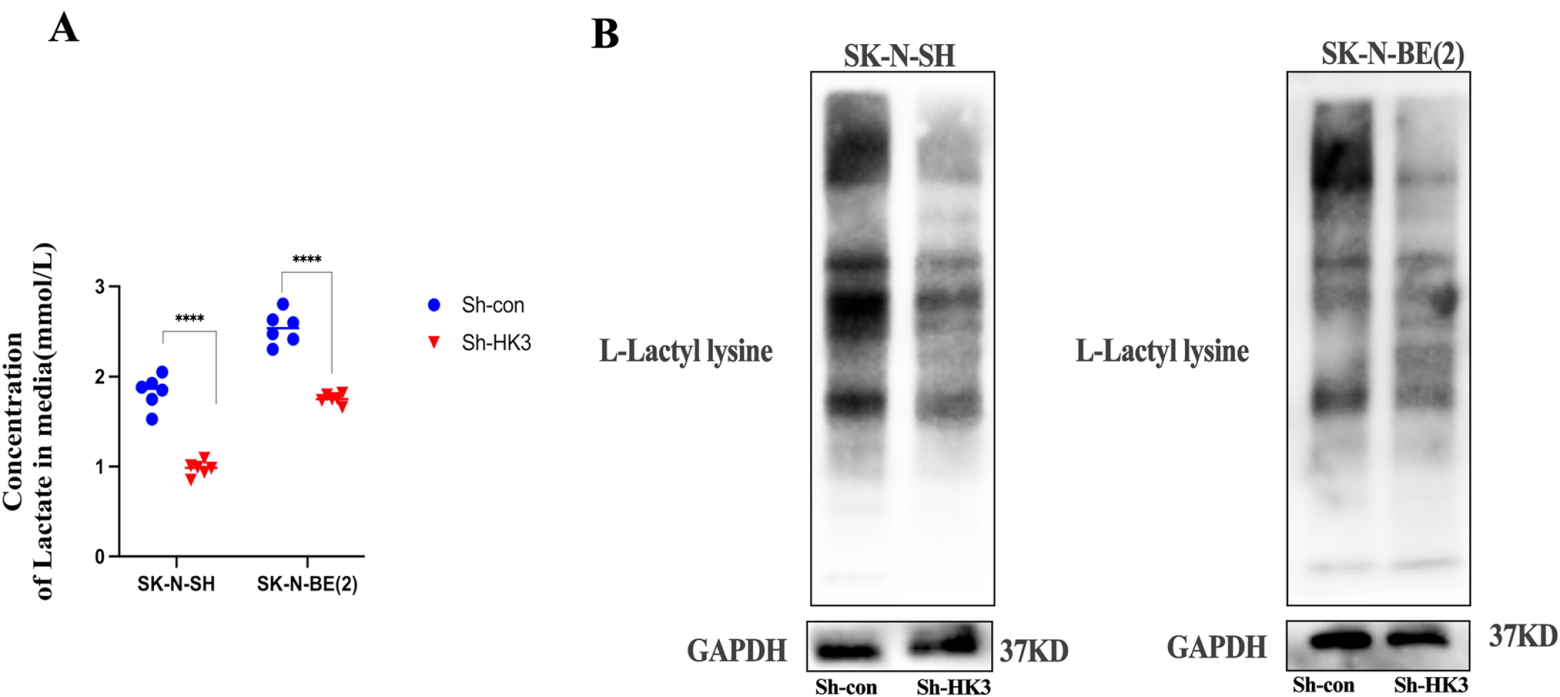
HK3 in neuroblastoma affects lactate secretion in the microenvironment and regulates histone lysine lactylation. **a** The concentration of Lactate in sh-con/sh-HK3 SK-N-SH and SK-N-BE(2) cells supernatant. **b** Western blot. l-Lactyl lysine expression of sh-con/sh-HK3 SK-N-SH and SK-N-BE(2) cells (****p < 0.0001)

Our data showed that levels of the histone lactylation protein were significantly reduced in SK-N-SH and SK-N-BE(2) cells after the knockdown of HK3 expression (Fig. 5b). Besides, the concentration of lactate in the culture supernatant of SK-N-SH/SK-N-BE(2) cells was significantly reduced after HK3 knockdown and thus we can deduce that HK3 attenuates the level of lactate and protein lactylation in neuroblastoma cells (Fig. 5a). Therefore, it is demonstrated that lactate secretion and histone lactylation, accompanied by M2 macrophage polarization in the NB microenvironment, can be regulated by HK3.

### HK3 regulates macrophage recruitment and M2-like macrophage polarization through CXCL14

**Fig. 6:**
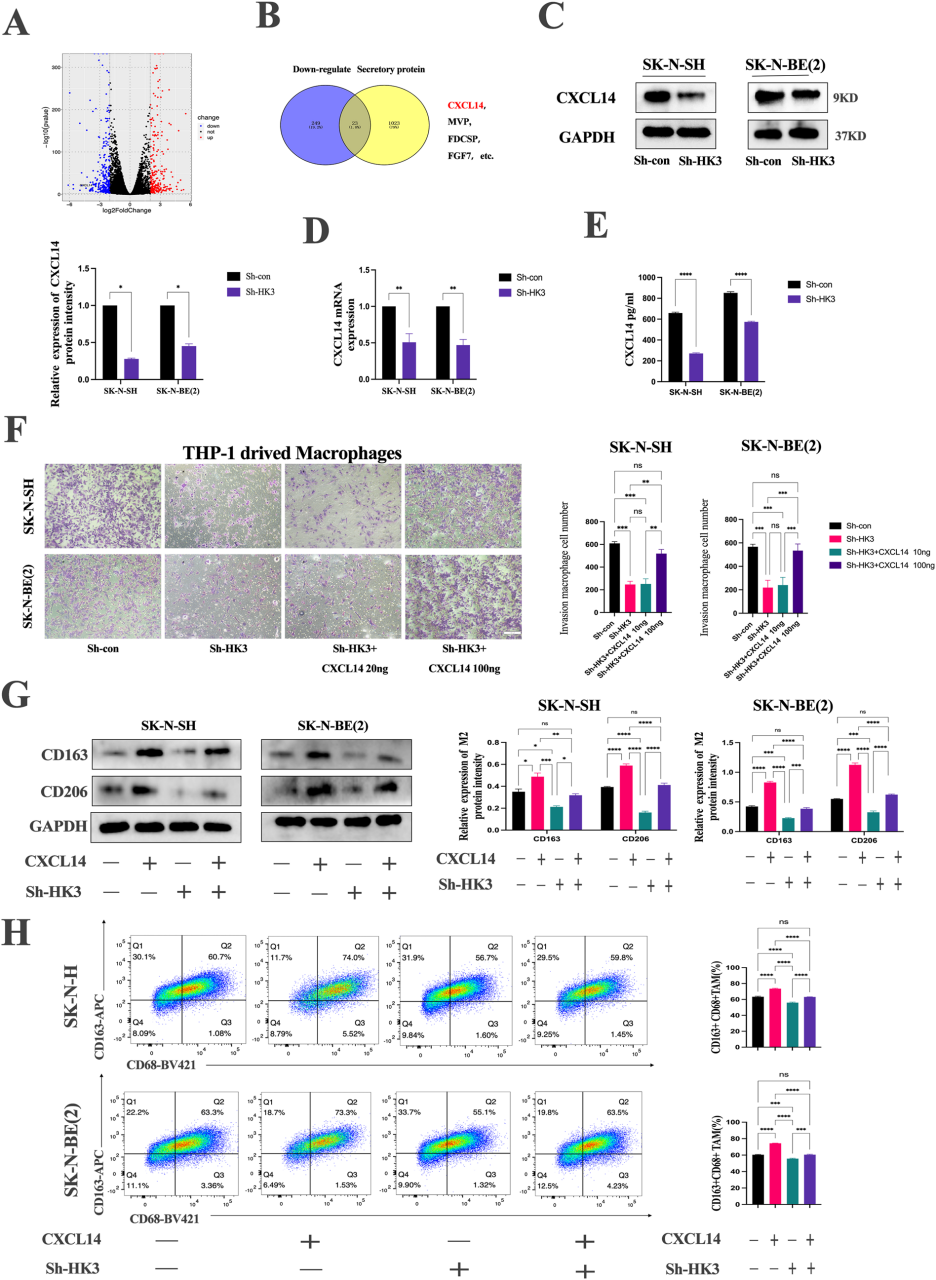
HK3 regulates macrophage recruitment and M2-like macrophage polarization through CXCL14. **a** A volcano plot of Differential Expression Analysis (DEG) in sh-con/sh-HK3 SK-N-SH cells. **b** The Venn diagram of down-regulated genes and secretory protein. **c** Western blot. The expression of CXCL14 protein in sh-con/sh-HK3 SK-N-SH and SK-N-BE(2) cells. **d** RT-PCR. The expression of CXCL14 mRNA in sh-con/sh-HK3 SK-N-SH and SK-N-BE(2) cells. **e** Elisa assay. The expression of CXCL14 protein in sh-con/sh-HK3 SK-N-SH and SK-N-BE(2) cells supernatant. **f** Macrophage migration assay. The migration and invasion ability of M2 macrophage cells after co-culturing with sh-con, sh-HK3, sh-HK3 with 20 ng/ml CXCL14, and sh-HK3 with 100 ng/ml CXCL14 SK-N-SH and SK-N-BE(2) for 48 h, scale bar = 100 μm. **g** Western blot. The expression of CD163, CD206, and GAPDH protein in macrophage cocultured with sh-con/sh-HK3 SK-N-SH and SK-N-BE(2) cells s group with or without 100 ng/ml CXCL14. **h** Flow cytometry. The dual expression of CD163 and CD68 of macrophage cocultured with sh-con/sh-HK3 SK-N-SH and SK-N-BE(2) cells group with or without 100 ng/ml CXCL14 (ns, no significance; *p < 0.05; **p < 0.01; ***p < 0.001, ****p < 0.0001)

The circulating lactate is directly derived from glycolysis, and lactate produced by tumors has been shown to drive M2-like polarization, leading to immune suppression. Numerous studies have focused on the direct effects of lactate on macrophages' M2 polarization, but the relevant mechanisms are not fully elucidated. To elucidate the mechanisms underlying the interaction between tumor cells and macrophages, RNA sequencing was performed to identify the 273 mRNAs downregulated in sh-HK3 SK-N-SH cells (Fig. 6a). Then, the secretory proteins database was obtained from Genecards (https://www.genecards.org/). 23 genes, including CXCL14, MVP, FDCSP, and FGF7, were identified by intersecting down-regulated genes and secreted proteins (Fig. 6b). It was found that CXCL14 (C-X-C Motif Chemokine Ligand 14), a secretory protein, has been reported to be involved in the homeostasis of monocyte-derived macrophages and also promotes immune cell infiltration in a variety of tumors [29, 30]. It was speculated that CXCL14 might play a role in the interaction between neuroblastoma and TAMs. Next, the expression of CXCL14 in sh-con and sh-HK3 SK-N-SH cells was validated. The q-PCR and Western Blot results indicated that CXCL14 was downregulated in sh-HK3 cells (Fig. 6c,d). ELISA also confirmed lower protein secretion of CXCL14 in the two HK3-knockout neuroblastoma cell lines (Fig. 6e).

To confirm the role of CXCL14 in TAM recruitment and polarization, an exogenous recombinant CXCL14 was added to the co-culture system. It was observed that 20 ng/ml recombinant CXCL14 protein had nearly no impact on the recruiting ability of macrophages in the HK3 knockdown group (Fig. 6f). However, 100 ng/ml recombinant CXCL14 protein significantly increased the recruiting ability of macrophages in the HK3 knockdown group, similar to that of the sh-con group (Fig. 6f). Western Blot and flow cytometry were performed to analyze the M2-TAMs markers in a co-culture system treated with recombinant 100 ng/ml CXCL14. The results demonstrated a significant increase in the M2 macrophage markers CD163 and CD206 in the sh-con/sh-HK3 group following the addition of recombinant CXCL14 (Fig. 6g, h). This result confirms that HK3 in neuroblastoma regulates the recruitment and polarization of M2-like macrophages by secreting CXCL14.

### Tumoral HK3 regulates the secretion and expression of CXCL14 via the PI3K-AKT pathway in NB

**Fig. 7:**
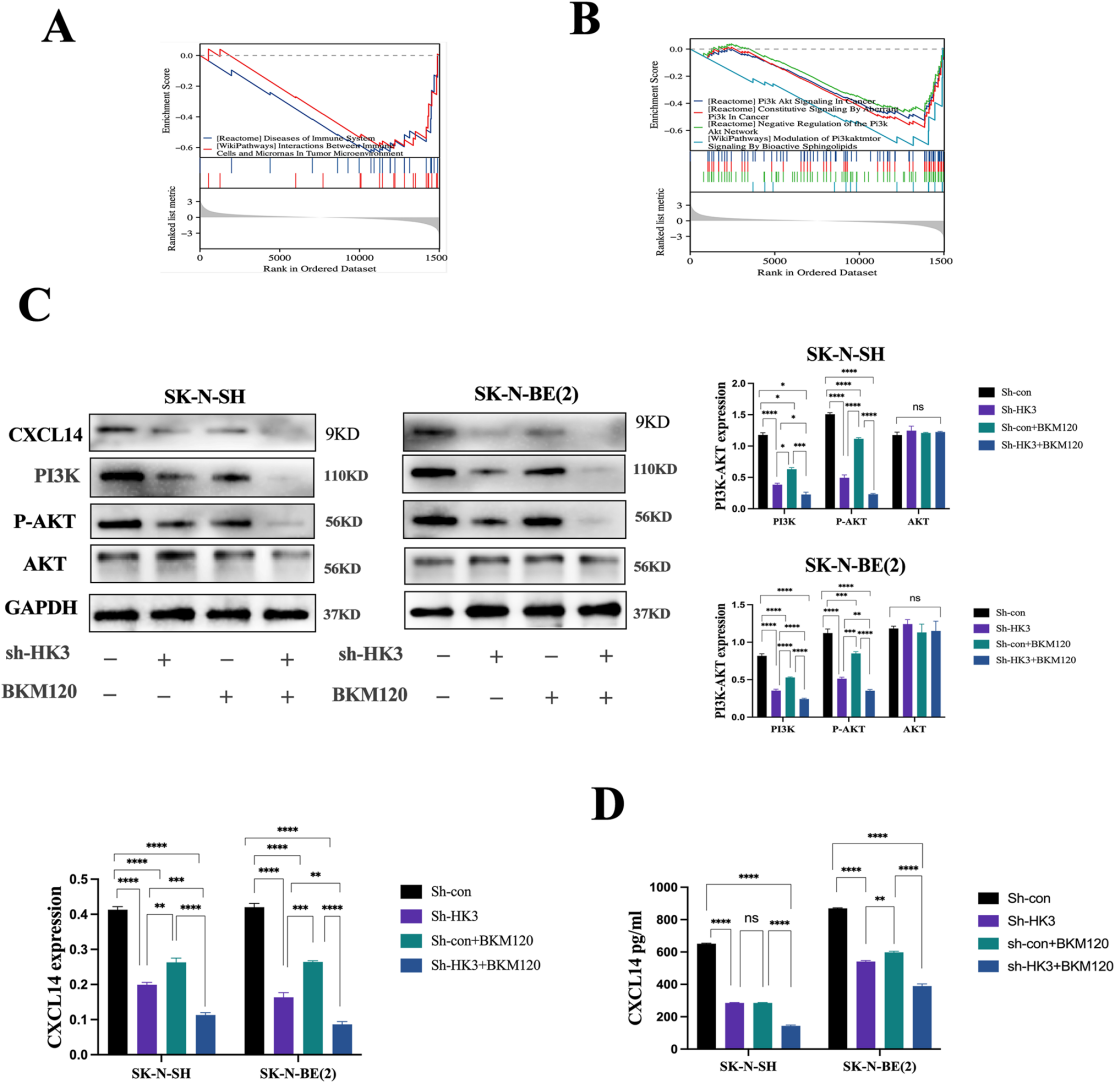
HK3 Orchestrates CXCL14 via PI3K-AKT Signaling to Propel Malignant Progression in NB. **a**–**b** Immune-related pathways, PI3K-AKT pathways were inhibited after the knockdown HK3 in SK-N-SH which was performed with the GSEA method. **c** Western blot. The expression of PI3K, P-AKT, PI3K, CXCL14, GAPDH protein in sh-con/sh-HK3 SK-N-SH and SK-N-BE(2) cells group with or without BKM120. **d** Elisa assay. The expression of CXCL14 protein in sh-con/sh-HK3 SK-N-SH and SK-N-BE(2) cells supernatant with or without BKM120 (ns, no significance; *p < 0.05; **p < 0.01; ***p < 0.001, ****p < 0.0001)

To ascertain how HK3 in neuroblastoma affects the functions of tumor-associated macrophages in the NB TME, RNA sequencing was conducted to screen the mRNA altered by knockdown of HK3 in SK-N-SH cells. Tumor microenvironment-related immune cell-related pathways were enriched and analyzed by GSEA, and PI3K-AKT signaling pathways were significantly downregulated at the same time (Fig. 7a, b). The phosphatidylinositol 3-kinase/Protein Kinase-B (PI3K/AKT) signaling pathway is one of the most activated pathways in most human cancers by promoting tumor cell survival, proliferation, metabolism, invasion, and angiogenesis [31]. The results indicate that the expression of PI3K p110α and p-Akt Ser473 proteins was decreased by the downregulation of HK3 (Fig. 7c). However, there was no significant change observed in the expression of AKT protein (Fig. 7c). To clarify the effect of one of the PI3K inhibitors BKM120 on the PI3K/Akt signaling pathway (SK-N-SH-0.99 μm, SK-N-BE(2)-1.2 μm), we examined the expression levels of PI3K/Akt signaling pathway related proteins. Western blot results showed that the expression of PI3K p110α and p-Akt Ser473 was significantly decreased both in sh-HK3 cells and the control group while there was no significant change in the expression of AKT after BKM120 intervention (Fig. 7c). Together, HK3-regulated the progression of neuroblastoma through the PI3K-Akt signaling pathway, and BKM120 effectively inhibited the activation of the PI3K/Akt pathway.

In preliminary experiments, it was identified that HK3 in neuroblastoma regulates the polarization and recruitment of M2-like macrophages through the secretion of CXCL14. Next, we aimed to determine whether CXCL14 is involved in the PI3K-AKT signaling pathway. The results show that the downregulation of HK3 decreased CXCL14 protein expression in SK-N-SH and SK-N-BE(2) (Fig. 7c, d). After adding PI3K inhibitor BKM120 0.99 μm to sh-con/sh-HK3 SK-N-SH, and 1.2 μm to sh-con/sh-HK3 SK-N-BE(2),the expression of CXCL14 protein was also decreased both in SK-N-SH and SK-N-BE (2) (Fig. 7c, d). This suggests that the expression of CXCL14 in neuroblastoma is also regulated by the PI3K-AKT signaling pathway. Therefore, HK3 exerts its influence on the interaction between neuroblastoma cells and M2-like macrophages by modulating CXCL14 via the PI3K-AKT signaling pathway.

### Knockdown of HK3 in M2 macrophages diminishes the proliferation, invasion, and migration capabilities of neuroblastoma cells

**Fig. 8:**
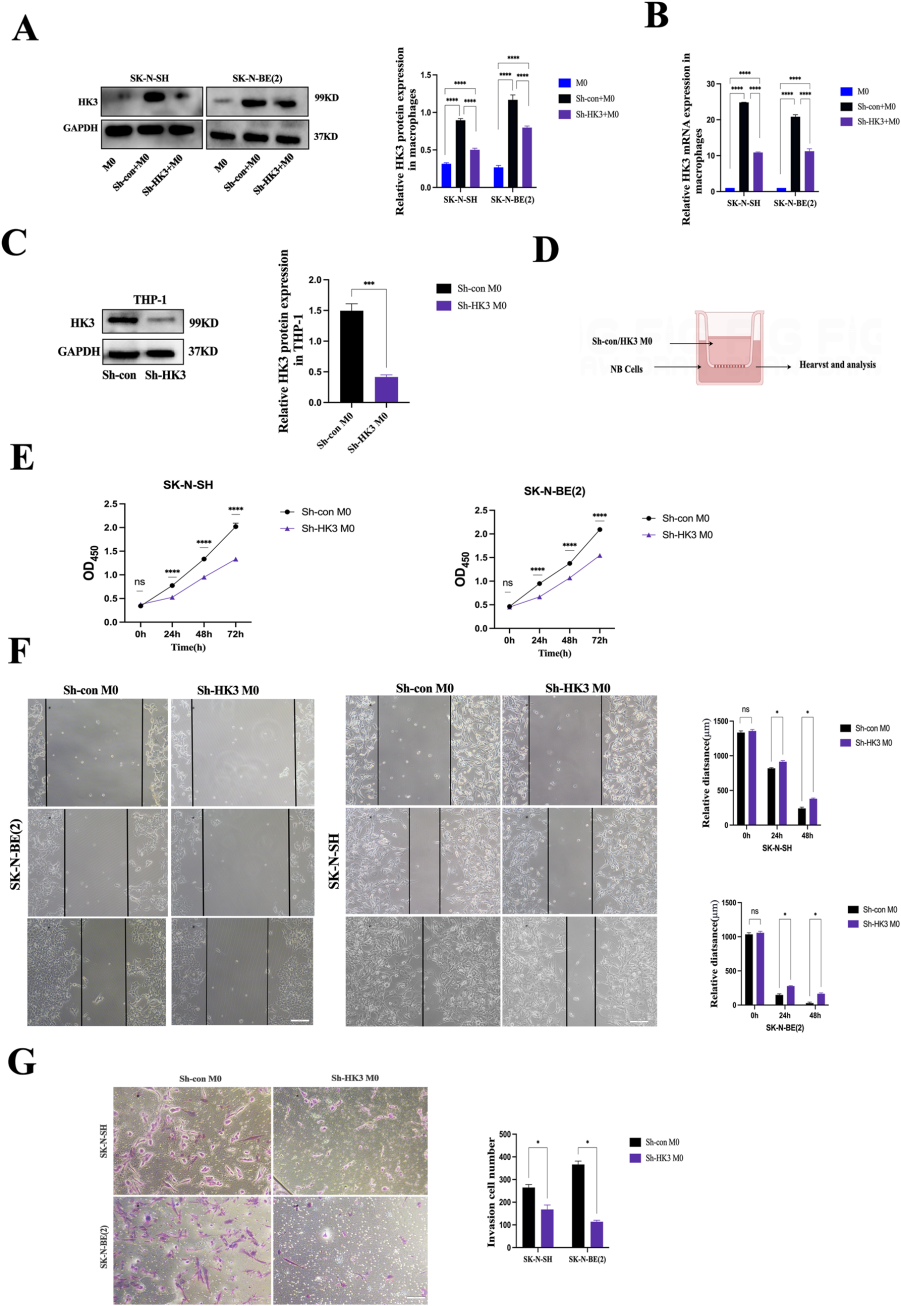
Knockdown of HK3 in M2 macrophages diminishes the proliferation, invasion, and migration capabilities of neuroblastoma cells. **a** The protein expression of HK3 in M0 and macrophage after co-culturing with sh-con/sh-HK3 SK-N-SH and SK-N-BE(2) for 48 h. **b** RT-PCR. The mRNA expression of HK3 in M0 and macrophage after co-culturing with sh-con/sh-HK3 SK-N-SH and SK-N-BE(2) for 48 h. **c** Schema for an in vitro model of transfected M0 cells co-cultured with NB cells. **d** Western blot. The expression of HK3 protein in sh-con/sh-HK3 THP-1. **e** CCK8 assay. Knock-down HK3 in macrophage decreased the proliferation of SK-N-SH and SK-N-BE(2) cells. **f** Wound-healing assay. The data showed that Knock-down HK3 in macrophage decreased the migration ability of SK-N-SH and SK-N-BE(2), scale bar = 100 μm. **g** Transwell assay. The data showed that Knock-down HK3 in macrophages decreased the invasion ability of SK-N-SH and SK-N-BE(2), scale bar = 100 μm (ns, no significance; *p < 0.05; **p < 0.01; ***p < 0.001, ****p < 0.0001)

Previous evidence suggests that tumor cell HK3 can reduce its secretion of lactate and cell communication factors, including CXCL14, thereby indirectly affecting the polarization of macrophages toward an M2-like phenotype and influencing tumor progression. Previous research has shown that macrophages co-cultured with tumor cells displaying M2-like phenotypes exhibited HK3 overexpression. This observation prompts speculation regarding the potential involvement of HK3 in M2-like macrophages (Fig. 8a, b). To investigate the function of HK3 in M2-TAMs, the sh-HK3-3 sequence, which had demonstrated the highest knockdown efficiency in preliminary experiments, was utilized to stably transfect sh-HK3 into THP-1 cells. Its effects on NB cells were then investigated. First, the transfection efficiency was confirmed by western blot (Fig. 8c). Next, a co-culture system was established with neuroblastoma cells and THP-1-derived macrophages knocked down by HK3. Subsequently, the impact of HK3 in M2-like macrophages on the biological behavior of neuroblastoma in vitro was explored (Fig. 8d).

Using the CCK8 assay, it was observed that M2-TAMs with low HK3 expression cause a decrease in the proliferation of two neuroblastoma cell lines (Fig. 8e). Next, using wound-healing and transwell assays, we observed that HK3-low macrophages markedly reduced the migration and invasion abilities of NB cells (Fig. 8f, g). Therefore, it was found that HK3 is also implicated in the impact of macrophages on tumor cells. Specifically, HK3 in M2-TAMs was discovered to play a role in the macrophage-mediated effects on tumor cells. Knockdown of HK3 in M2 macrophages was observed to diminish the proliferation, invasion, and migration capabilities of neuroblastoma cells.

## Discussion

Neuroblastoma (NB), the most common extracranial solid tumor in infants, originates from primitive sympathetic nervous precursor cells [32]. The survival rate for neuroblastoma (NB) patients stands at approximately 60%. However, this figure dwindles to 10–15% when low-intensity chemotherapy is applied to infants under three years of age. Consequently, neuroblastoma emerges as a predominant factor in pediatric solid tumor-related fatalities [33]. Distinct from adult tumors, pediatric tumor immune microenvironments are dominated by innate immunity, with a significant role played by tumor-associated macrophages (TAMs) [34, 35]. Notably, M2-like macrophages in neuroblastoma are linked to chemotherapy resistance and poor prognosis.

Targeting TAMs appears to be a promising therapeutic strategy for pediatric solid tumors. Tumor cells can attract and polarize macrophages to drive tumor progression, with many genes involved in this process. For instance, the interplay between macrophages and glioma cells through a positive feedback loop via the IFN-γ-IRF2-ARPC1B axis regulates tumor progression [21]. PCSK9 plays a crucial role in the progression and metastasis of colon cancer by regulating tumor cell EMT and the PI3K/AKT signaling pathway, and by mediating MIF and lactate levels to control macrophage phenotype polarization [36]. Therefore, we hypothesize that a similar regulatory mechanism between macrophages and NB cells in neuroblastoma.

Metabolic reprogramming can accelerate tumor cell proliferation and growth by regulating energy metabolism, such as tumor cells preferentially utilizing the glycolytic pathway (Warburg effect) to rapidly provide ATP for their development, growth, and metastasis [16]. As the rate-limiting enzyme driving glycolysis, hexokinase plays a crucial role in tumor tissue glycolysis [18]. Compared to HK1 and HK2, HK3 is less reported in tumors. However, recent studies have gradually revealed the role of HK3 in tumors. Studies show that HK3 often collaborates with immune cells to promote tumor progression. HK3 is correlated with immune infiltrates and predicts response to immunotherapy in non-small cell lung cancer [37]. In glioma, HK3 enhances immune cell infiltration and malignancy [38]. In our study, it was initially observed that overexpression of HK3 correlated with a poor prognosis in neuroblastoma, with HK3 expression consistently heightened in NB INSS IV. M2-like macrophages are highly infiltrated in neuroblastoma tumors and are strongly associated with HK3, as confirmed in bioinformatic analysis and immunohistochemistry or Western blotting of clinical tissues. Immunofluorescence colocalization further demonstrates their close relationship, leading us to speculate that they may interact through some underlying mechanism in NB progression.

Hence, the primary focus of our investigation was to elucidate the role of HK3 in NB cells. The experiments in vitro and in vivo both illuminated the tumor-promoting role of HK3. This indicates that at the level of NB cells, HK3 can regulate the biological behavior of the tumor. To validate the correlation between HK3 in neuroblastoma and macrophages, an in vitro co-culture system of neuroblastoma with THP-1-derived macrophages was established. THP-1 cells are a widely used cellular tool in immunology and inflammation research in vitro [39]. It was observed that the recruitment and polarization abilities of M2 macrophages were diminished following co-culture with HK3-knockdown neuroblastoma cells. In in vivo experiments, to eliminate the impact of intrinsic macrophages in mice, clodronate liposomes were utilized to deplete macrophages. The results revealed that mice with macrophage depletion and solely implanted with HK3-knockdown neuroblastoma exhibited the smallest tumor size and weight, the lowest malignancy within the tumor, and the least infiltration of M2-like macrophages. In contrast, the group with macrophages co-cultured with control neuroblastoma cells exhibited the largest tumor volume and weight, the highest malignancy, and the most infiltration of M2-like macrophages, with even more tumor metastasis sites. This suggests a significant regulatory role of HK3 in neuroblastoma tumorigenic capacity when co-cultured with tumor-associated macrophages, particularly those exhibiting an M2-like phenotype.

HK3 is a subtype of hexokinase involved in the first step of glucose metabolism, lactate is a direct by-product of glycolysis, which has been widely demonstrated to be directly involved in tumor progression by M2 macrophage polarization in tumor microenvironment [16, 28, 40, 41]. Histone lysine lactylation (Kla) was identified as a novel post-modification (PTM) [27]. Numerous studies have demonstrated that lactate promotes the polarization of macrophages toward an M2-like phenotype through the lactylation of histone lysines [27, 42–44]. Therefore, it was crucial for us to determine whether HK3, a key gene in glycolysis, is involved in regulating lactate production in NB. The results showed that knocking down HK3 significantly decreased lactate secretion in NB and reduced the level of histone lysine lactylation in NB.

In the tumor microenvironment characterized by high lactate and low pH, tumor cells frequently interact with tumor-associated macrophages (TAMs) through molecular mediators such as cytokines, chemokines, and exosomes. For instance, FOXO1 promotes macrophage recruitment and M2-like macrophage polarization by enhancing the secretion of CSF-1 (colony-stimulating factor 1) and CCL20 (chemokine ligand 20) in esophageal cancer [45]. In our experiment, we hypothesized that tumor cells with knocked-down HK3 could reduce M2-like macrophage characteristics, possibly through intermediary-secreted mediators. Transcriptome sequencing and GSEA enrichment analysis revealed a significant reduction in CXCL14 expression in neuroblastoma cells with knocked-down HK3, accompanied by a notable decrease in secreted CXCL14. CXCL14 mainly contributes to the regulation of immune cell migration [30]. Studies have shown that this cytokine is involved in the in vivo balance of monocyte-derived macrophages [29]. Similarly, CXCL14 enhances the propensity of cancer cells toward bone and/or recruits bone marrow cells around metastatic cancer cells to promote bone metastasis in lung cancer [46]. CXCL14 also promotes tumor lymphocyte infiltration in oral squamous cell carcinoma [47]. In our experiments, following the exogenous addition of 100 ng/ml CXCL14 to the in vitro co-culture system, we observed a notable increase in the recruitment and polarization levels of M2-like macrophages in the HK3 knockdown group. This suggests that CXCL14 serves as a downstream molecule in neuroblastoma (NB), and HK3 regulates the recruitment and polarization of M2-like macrophages through CXCL14.

As previously reported in many types of cancer, including neuroblastoma, the PI3K/AKT signal transduction pathway is closely correlated with tumor proliferation and patient survival [31]. In this experiment, transcriptome sequencing and GSEA enrichment analysis were performed on SK-N-SH neuroblastoma cells with knocked-down HK3 and the control group. The enrichment analysis revealed PI3K-AKT signaling pathway was activated. Subsequent verification demonstrated that HK3 activation in neuroblastoma triggers the PI3K-AKT signal transduction pathway, thereby influencing the expression of CXCL14 in neuroblastoma. Consequently, HK3 modulates the interaction between neuroblastoma and M2-like macrophages by secreting CXCL14 through the PI3K/AKT signaling pathway.

Previous experiments have shown that tumor cell HK3 can decrease its secretion of lactate and CXCL14, indirectly impacting the polarization of macrophages toward an M2-like phenotype, thereby influencing tumor progression. During this process, we examined the expression of HK3 in M2-like macrophages and found it to be significantly higher than in M0 macrophages, suggesting a potential role for HK3 in M2 macrophages. Therefore, HK3 was knocked down in THP-1 cells and subsequently applied to NB cells in a co-culture system. We observed that the decreased expression of HK3 in M2 macrophages indirectly led to a reduction in the proliferation, invasion, and migration abilities of NB tumor cells. Consequently, it is believed that M2-like macrophages with high expression of HK3 can regulate the malignant biological behavior of neuroblastoma, and they also hold potential as therapeutic targets for NB. However, further investigation is required to elucidate the underlying mechanisms.

In summary, our study results indicate that HK3 serves as a regulatory factor in the NB-TAM interaction, modulating the crosstalk between neuroblastoma cells and M2-like macrophages. The dual role of HK3 in coordinating neuroblastoma malignancy underscores its potential as a multifaceted target for tumor therapy and diagnosis. Elucidating the significant role of HK3 in the pathogenesis of neuroblastoma not only deepens our understanding of the dynamics of the TME but also paves the way for new intervention strategies, ultimately improving clinical management and prognosis for neuroblastoma patients.

### Supplementary Information

Below is the link to the electronic supplementary material.

Supplementary Figure 1. Overexpression of HK3 indicated a poor prognosis in neuroblastoma patients and the landscape of immune infiltration in NB. (A-C) Kaplan-Meier curves for eventfree survival probability in NRC-283(2888485) HK3(A), Kaplan-Meier curves for progressionfree survival probability in Kocak-476(ukv A24) HK3(B), Kaplan-Meier curves for relapsefree survival probability in Vesteeg-88 (205936-s-at) data set. (D) The landscape of immune infiltration in NB. (E) Kaplan-Meier curves for eventfree survival probability in NRC-283(2888485) HK3. (E) Kaplan-Meier survival analysis identified M2 macrophage as a stable unfavorable prognostic factor in TARGET NB cohorts. (F) The correlation between HK3 and M2 macrophage in the TARGET database (r = 0.31)
Supplementary file1 (CSV 1451 KB)

Supplementary Figure 2. Expression of HK3 in neuroblastoma cell lines and validation of the efficiency of HK3 knockdown. (A) Expression of HK3 in neuroblastoma cell lines and Huevc. (B) RT-PCR. The mRNA expression of HK3 knockdown in SK-N-SH and SK-N-BE(2). (C) Western blot. The protein expression of HK3 knockdown in SK-N-SH and SK-N-BE(2)( ns, no significance；*p < 0.05; **p < 0.01; ***p < 0.001, ****p < 0.0001)
Supplementary file2 (TIF 4737 KB)

Supplementary Figure 3. The morphology of macrophage, validation of the efficiency of macrophage depletion, and PBS liposomes group statistics. (A)Microscopic morphology of THP-1 and different stage of macrophages. (B)Immunohistochemical staining of the macrophage marker F4/80 in mice kidney, lung, spleen, liver. (C)Macroscopic images of kidney and tumor tissues of 2 PBS liposome transplant groups (sh-HK3, sh-con). (D-E) Quantitative analysis of tumor size(D) and tumor weight (E) ( ns, no significance；*p < 0.05; **p < 0.01; ***p < 0.001, ****p < 0.0001)
Supplementary file3 (TIF 3278 KB)

The illustration of the regulating networks between NB cells and TAMs is based on HK3
Supplementary file4 (TIF 191995 KB)

Supplementary file5 (PNG 746 KB)

## Data Availability

The data supporting this study's findings are openly available in The TARGET database (https://ocg.cancer.gov/programs/target). For more details, upon a reasonable request, you can also approach the corresponding author at any time and request the data.
