# Supplementary figures and images for: Dual roles of HK3 in regulating the network between tumor cells and tumor-associated macrophages in neuroblastoma
Source: Cancer Immunol Immunother. 2024 May 7;73(7):122. doi: 10.1007/s00262-024-03702-9 (PMC11076449; doi:10.1007/s00262-024-03702-9)

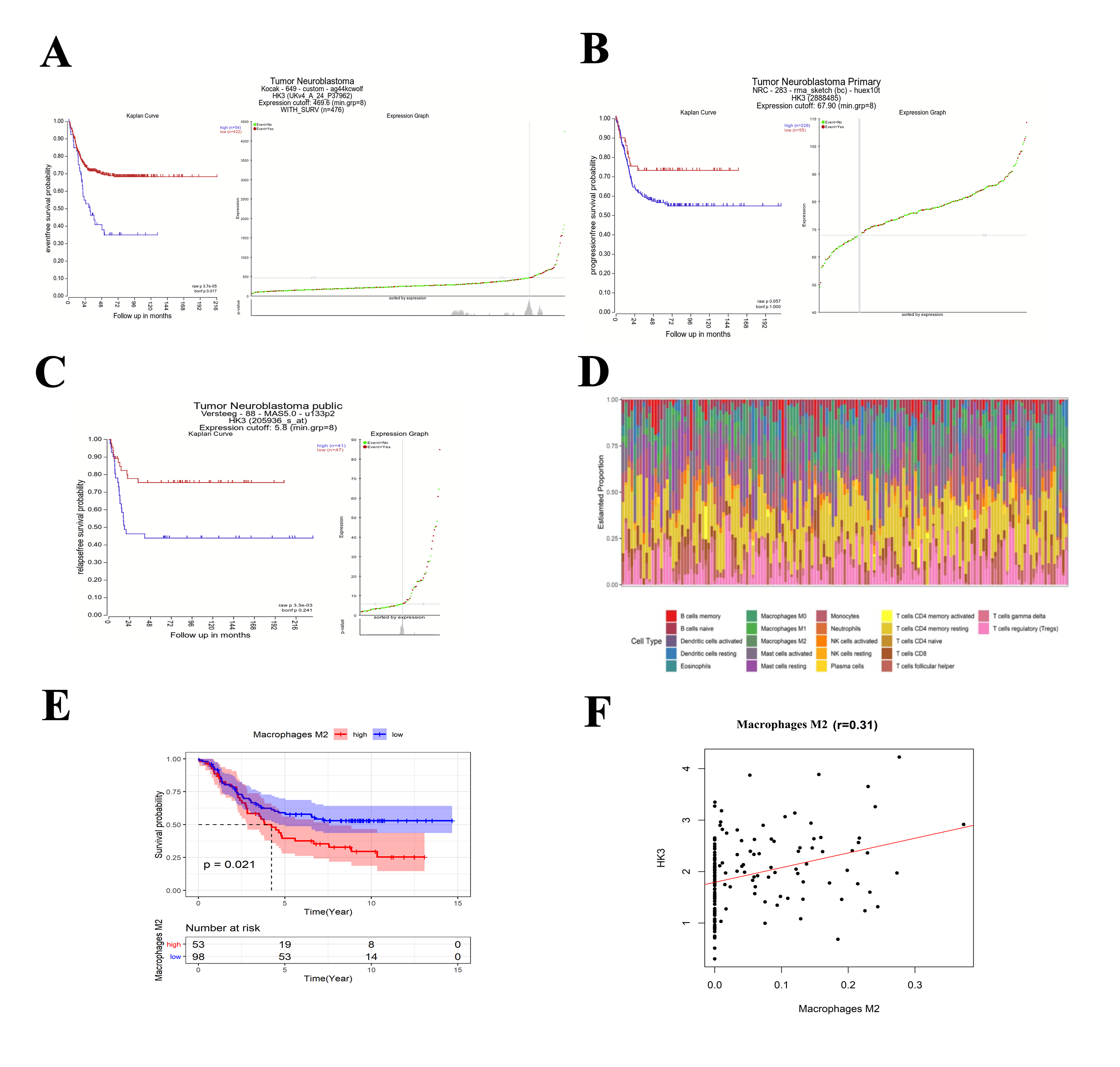

Supplement: Supplementary file 2 — Supplementary Figure 2. Expression of HK3 in neuroblastoma cell lines and validation of the efficiency of HK3 knockdown. (A) Expression of HK3 in neuroblastoma cell lines and Huevc. (B) RT-PCR. The mRNA expression of HK3 knockdown in SK-N-SH and SK-N-BE(2). (C) Western blot. The protein expression of HK3 knockdown in SK-N-SH and SK-N-BE(2)( ns, no significance；*p < 0.05; **p < 0.01; ***p < 0.001, ****p < 0.0001) Supplementary file2 (TIF 4737 KB) [file 262_2024_3702_MOESM2_ESM.tif]

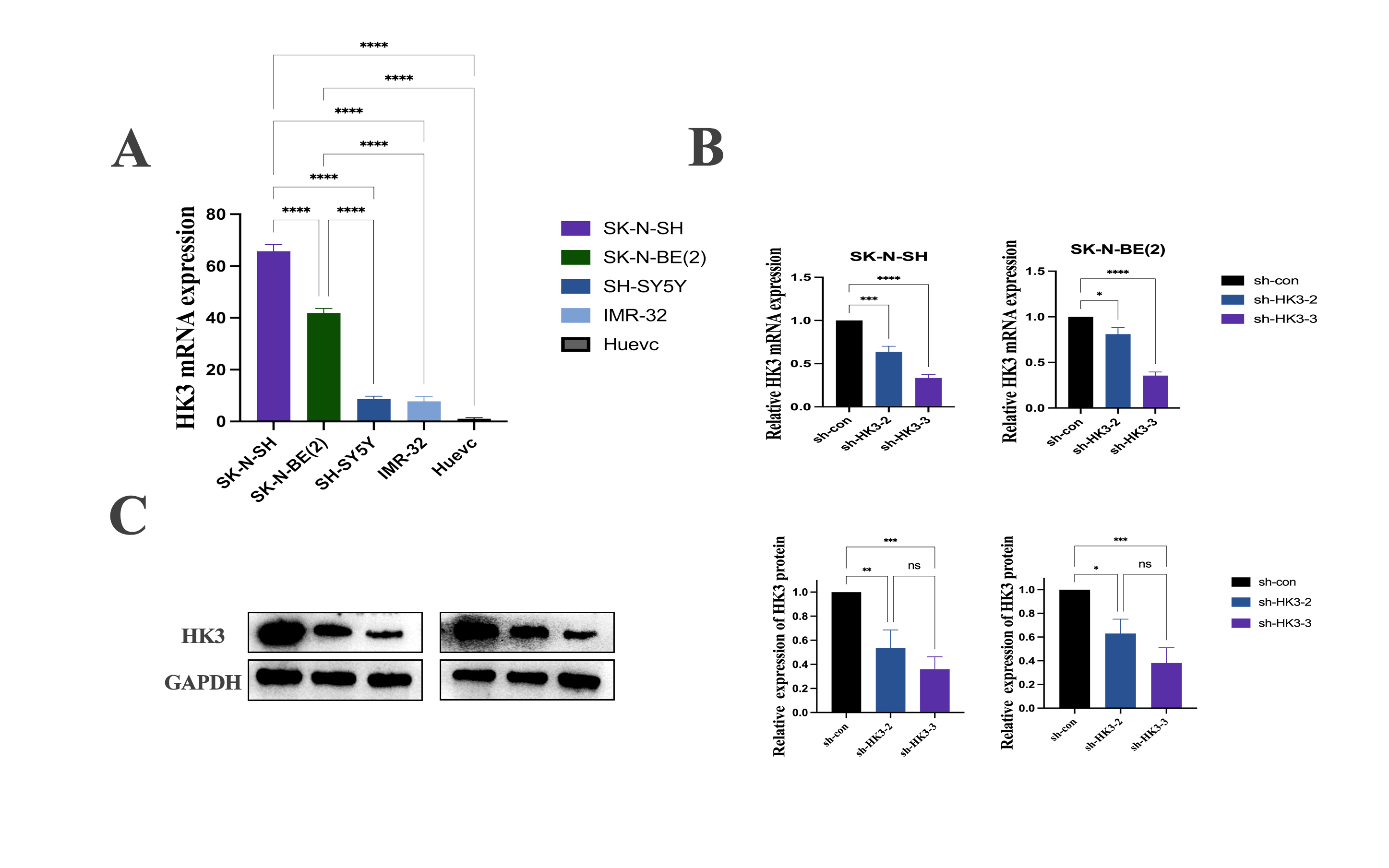

Supplement: Supplementary file 3 — Supplementary Figure 3. The morphology of macrophage, validation of the efficiency of macrophage depletion, and PBS liposomes group statistics. (A)Microscopic morphology of THP-1 and different stage of macrophages. (B)Immunohistochemical staining of the macrophage marker F4/80 in mice kidney, lung, spleen, liver. (C)Macroscopic images of kidney and tumor tissues of 2 PBS liposome transplant groups (sh-HK3, sh-con). (D-E) Quantitative analysis of tumor size(D) and tumor weight (E) ( ns, no significance；*p < 0.05; **p < 0.01; ***p < 0.001, ****p < 0.0001) Supplementary file3 (TIF 3278 KB) [file 262_2024_3702_MOESM3_ESM.tif]

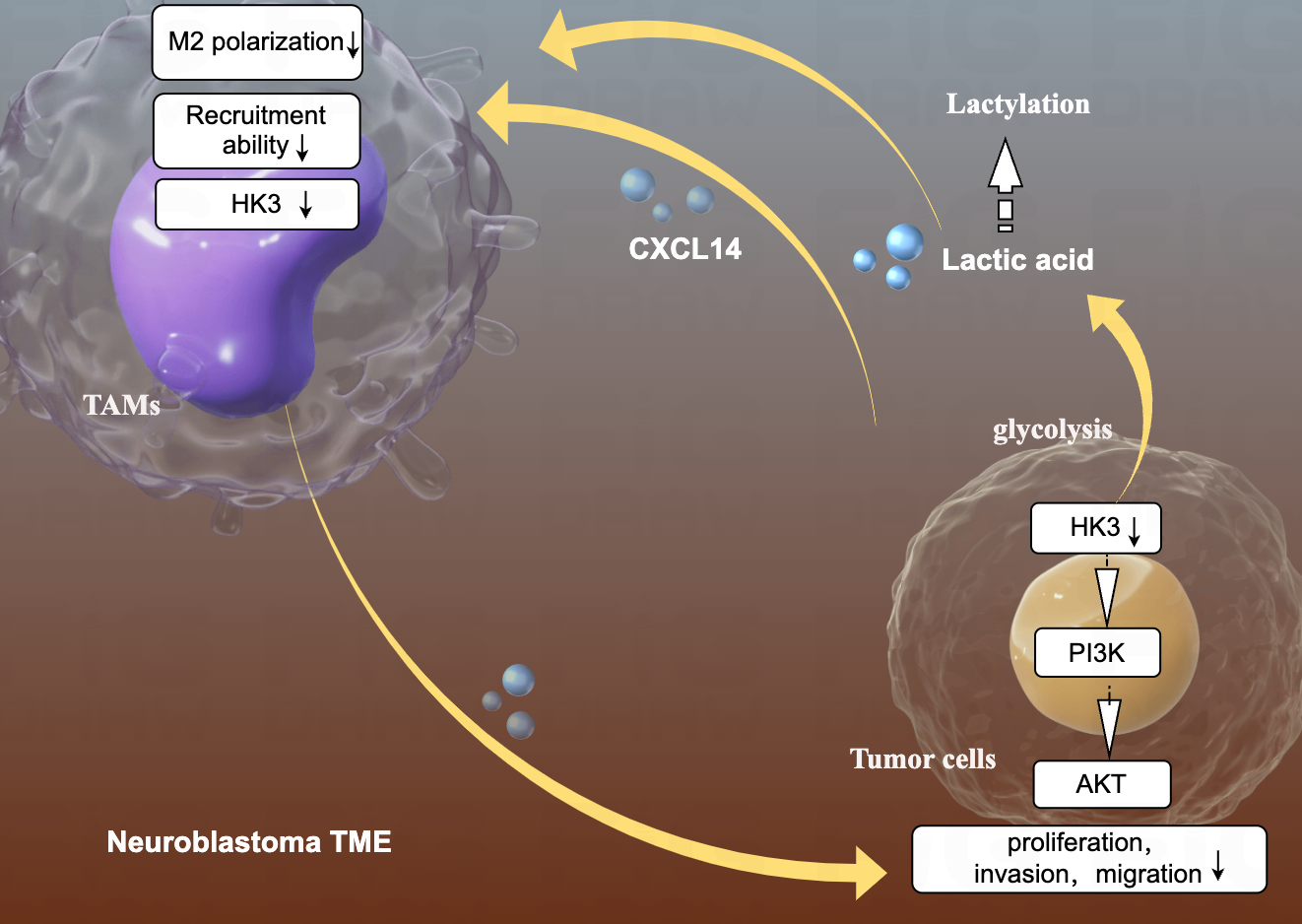

Supplement: Supplementary file 5 — Supplementary file5 (PNG 746 KB) [file 262_2024_3702_MOESM5_ESM.png]
